# Author Correction: Jawsamycin exhibits in vivo antifungal properties by inhibiting Spt14/Gpi3-mediated biosynthesis of glycosylphosphatidylinositol

**DOI:** 10.1038/s41467-020-17748-7

**Published:** 2020-07-31

**Authors:** Yue Fu, David Estoppey, Silvio Roggo, Dominik Pistorius, Florian Fuchs, Christian Studer, Ashraf S. Ibrahim, Thomas Aust, Frederic Grandjean, Manuel Mihalic, Klaus Memmert, Vivian Prindle, Etienne Richard, Ralph Riedl, Sven Schuierer, Eric Weber, Jürg Hunziker, Frank Petersen, Jianshi Tao, Dominic Hoepfner

**Affiliations:** 1 Genomics Institute of the Novartis Research Foundation, 10675 John Jay Hopkins Drive, San Diego, CA 92121 USA; 2 Novartis Institutes for BioMedical Research, Novartis Pharma AG, Forum 1 Novartis Campus, CH-4056 Basel, Switzerland; 3 The Lundquist Institute for Biomedical Innovations at Harbor-University of California at Los Angeles (UCLA) Medical Center, Torrance, CA 90502 USA; 4 David Geffen School of Medicine at UCLA, Los Angeles, CA 90095 USA

**Keywords:** Target identification, Antifungal agents, Fungi, Pathogens

Correction to: *Nature Communications*10.1038/s41467-020-17221-5, published online 7 July 2020.

The original version of this Article omitted the following from the Acknowledgements:

We also thank the Novartis Chemical & Pharmaceutical Profiling & DMPK teams for their support on evaluation of in vivo efficacy of Jawsmycin.

This has now been corrected in both the PDF and HTML versions of the Article.

