# Supplementary material for: Jawsamycin exhibits in vivo antifungal properties by inhibiting Spt14/Gpi3-mediated biosynthesis of glycosylphosphatidylinositol
Source: Nat Commun. 2020 Jul 7;11:3387. doi: 10.1038/s41467-020-17221-5 (PMC7341893; doi:10.1038/s41467-020-17221-5)
Supplement: Supplementary file 1 — Supplementary Information [file 41467_2020_17221_MOESM1_ESM.pdf]

Supplementary Information

Jawsamycin exhibits *in vivo* antifungal properties by inhibiting Spt14/Gpi3-mediated biosynthesis of glycosylphosphatidylinositol

Yue Fu, David Estoppey, Silvio Roggo, Dominik Pistorius, Florian Fuchs, Christian Studer et al.,

Page 2 -6: Supplementary Figures

Page 2: Supplementary Figure 1. Dose-response testing of reporter assay paradigm.

Page 3: Supplementary Figure 2. Validation of chemogenomic profiling results by single-strain testing.

Page 4: Supplementary Figure 3. Multiple sequence alignment.

Page 6 -18: Supplementary Methods

Page 6: Compound purification methods

Page 6 -19: Synthesis and characterization of compounds related to this study

Supplementary Figures

Supplementary Figure 1. Dose-response testing of reporter assay paradigm.

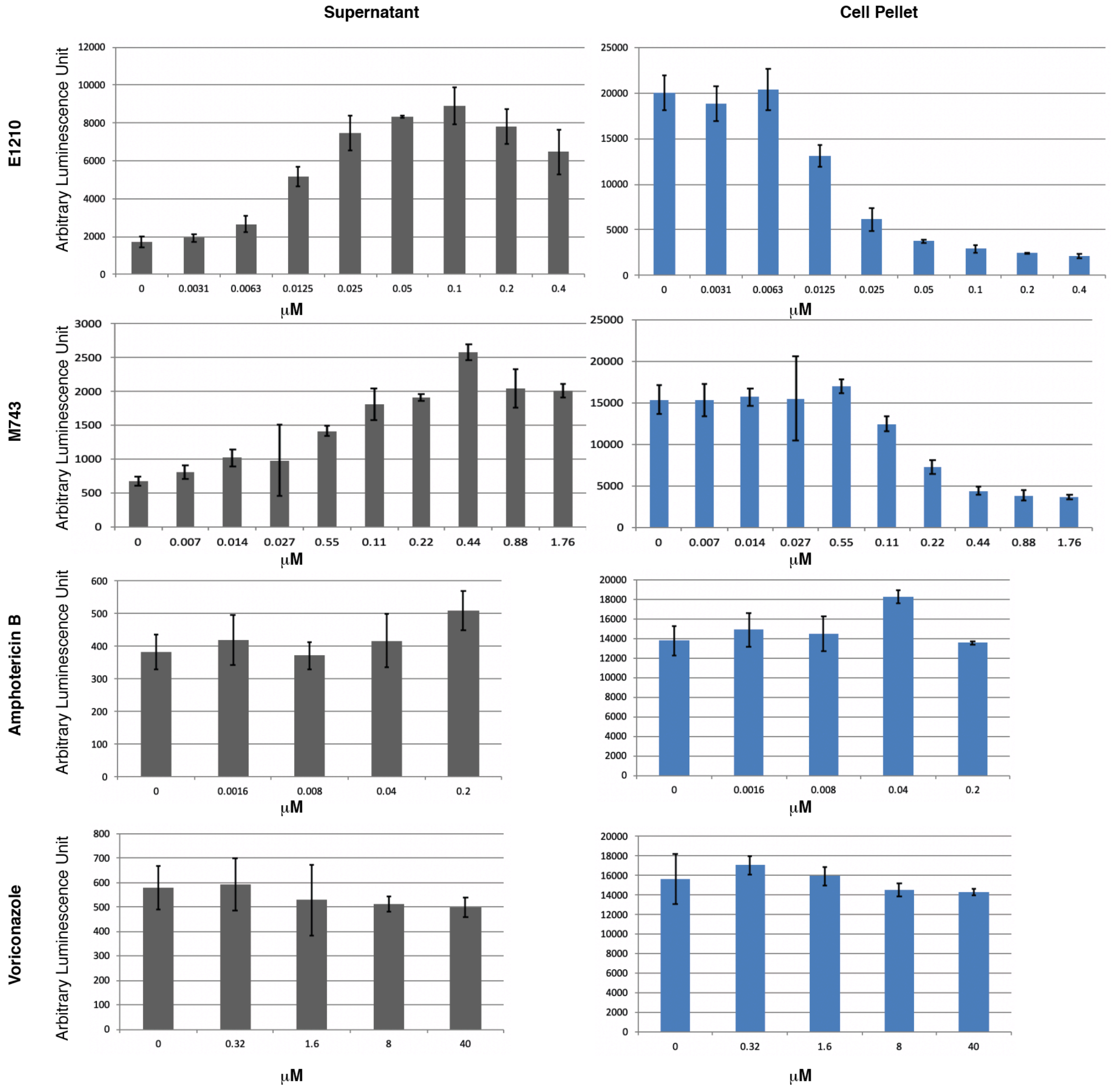

Dose-response data generated during assay development. Known GPI inhibitors (top two rows) but not plasma membrane and cell-wall inhibitors (bottom two rows) lead to increase of luminescence signal in supernatant and reduction of signal of cell pellet.

Supplementary Figure 2. Validation of chemogenomic profiling results by single-strain testing.

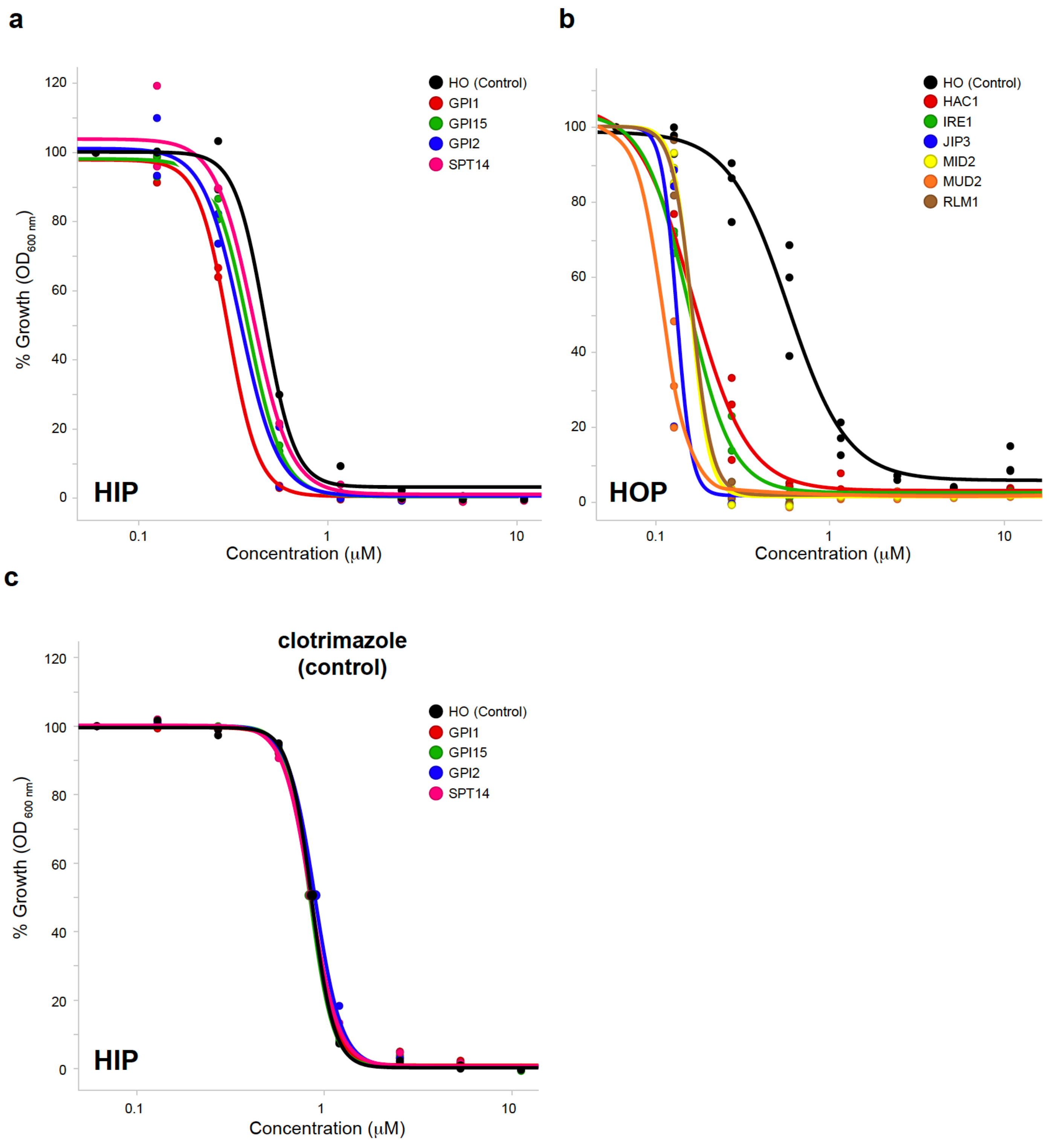

a) Single strain validation of heterozygous deletion strain observed in the HIP experiment shown in Figure 2a. b) Single strain validation of homozygous deletion strains observed in the HOP experiment shown in Figure 2b. c) Effects observed with jawsamycin were found to be specific as none of the heterozygous strains scored with the ergosterol inhibitor clotrimazole. N = 2 data was recorded for validation of the HIP experiment (panel a, c), n = 3 data was recorded for the validation of the HOP experiment (panel b), curves represent the calculated mean.

Supplementary Figure 3. Multiple sequence alignment.

```
A. nidulans         ------------------------------------------------------------
F. oxysporum        ------------------------------------------------------------
S. cerevisiae       ------------------------------------------------------------
C. albicans         ------------------------------------------------------------
R. delemar          ------------------------------------------------------------
C. neoformans       MWKVIYYLNLALYTVLLLTTSFIAVLIAIVCSLTGRRLNTNYFVARTFYHFAGPILGWKF
A. flavus.          ------------------------------------------------------------
A. fumigatus        ------------------------------------------------------------
M. circinelloides   ------------------------------------------------------------

A. nidulans         ------------------------------------------------------------
F. oxysporum        ------------------------------------------------------------
S. cerevisiae       ------------------------------------------------------------
C. albicans         ------------------------------------------------------------
R. delemar          ------------------------------------------------------------
C. neoformans       QVEGEQYLWELSGEHGGGKAGEKGRSMVMVGNHQSFVDILYLGRIFPKHAAIMAKKSIQW
A. flavus.          ------------------------------------------------------------
A. fumigatus        ------------------------------------------------------------
M. circinelloides   ------------------------------------------------------------

A. nidulans         ------------------------------------------------------------
F. oxysporum        ------------------------------------------------------------
S. cerevisiae       ------------------------------------------------------------
C. albicans         ------------------------------------------------------------
R. delemar          ------------------------------------------------------------
C. neoformans       IPGLGWFMMMSGTVFINRSNNKSAIASLQHAGEEMKRKRISLWIFPEGTRHNTPEPELLN
A. flavus.          ------------------------------------------------------------
A. fumigatus        ------------------------------------------------------------
M. circinelloides   ------------------------------------------------------------

A. nidulans         ------------------------------------------------------------
F. oxysporum        ------------------------------------------------------------
S. cerevisiae       ------------------------------------------------------------
C. albicans         ------------------------------------------------------------
R. delemar          ------------------------------------------------------------
C. neoformans       FKKGAFYLAVQAGVPIVPVVCENYNHLFNGKSHFRRGTLRIKVLPPIPTTGLTTADVPNL
A. flavus.          ------------------------------------------------------------
A. fumigatus        ------------------------------------------------------------
M. circinelloides   ------------------------------------------------------------

A. nidulans         ------------------------------------------------------------
F. oxysporum        ------------------------------------------------------------
S. cerevisiae       ------------------------------------------------------------
C. albicans         ------------------------------------------------------------
R. delemar          ------------------------------------------------------------
C. neoformans       IEKTRNAMLETLREISTPSQSTSQAGSPDPLLGRPGRERENYYTSGSPAPPEGVSSAAEI
A. flavus.          ------------------------------------------------------------
A. fumigatus        ------------------------------------------------------------
M. circinelloides   ------------------------------------------------------------

A. nidulans         ------------------------------------------MPYNIAMVSDFFFPQPGG
F. oxysporum        ------------------------------------------------------------
S. cerevisiae       ------------------------------------------MGFNIAMLCDFFYPQLGG
C. albicans         ------------------------------------------MGYNIAMVTDFFYPQPGG
R. delemar          ---------------------------------------------------------MGG
C. neoformans       GAEEEAEAAVEDAIGREEADNGERHVHVVGKNDRGDETMSSPKRLAIAMVSDFFFPVIGG
A. flavus.          ----------------------------MASFTSKPDDFPKILKGRRVLLTTESLGPVNG
A. fumigatus        ---------------------------------MDRESFPQSLKGKKVLLTTESLGPVNG
M. circinelloides   --------------------------------------------MRIAIITENFLPKVDG

A. nidulans         VESHIYQLSTKLIDRGHKVIIITHAYKGRTGVRYLTNGLKVYHVPFLVIYRETTMPTVFS
F. oxysporum        ------------------------------------------------------------
S. cerevisiae       VEFHIYHLSQKLIDLGHSVVIITHAYKDRVGVRHLTNGLKVYHVPFFVIFRETTFPTVFS
C. albicans         VEFHVYHLSQKLIELGHSVVIITHNYSSRNGVRVLTNGLKVYYVPLWVIYRSSVFPTVFS
R. delemar          VESHLYELSQRLIQRGHKVIIVTHAYGNRTGVRYLTNGLKVYYVPAKVIYSEATLPTIYG
```

```
C. neoformans       VEGHIYSLSVELMRRGHKVIVITHSHPDRLGIHYLGPSLKVYYLPYLPIASSASLPNFLL
A. flavus.          VSRTTLSLVEYLRRNGVDLAVVAPQYQGFRYQAQDAADCRIPGYPLPYNPDLTIVYPFRL
A. fumigatus        VSRTTGSLIDYLRRNEVDLMVVAPKFAGAQQAEQPEANLRLPGYPLPYNPDLTLVYPFRL
M. circinelloides   VTRTLARLLEHLSKMGHQVLLLGPETNMTTYA--GANLVGTYGIPFFLYPELKFNFWRPK


A. nidulans         FFPIFRNIVIREQIQIVHGHQSLSSFCHEAILHARTMGLRTAFTDHSLFGFADAGSILTN
F. oxysporum        -----------------------------------MGLRTVFTDHSLFGFADAGTILTN
S. cerevisiae       TFPIIRNILLREQIQIVHSHGSASTFAHEGILHANTMGLRTVFTDHSLYGFNNLTSIWVN
C. albicans         CFPILRNIFIRENIEIIHGHGSFSTLCHEAILHGRTMGLKTVFTDHSLFGFAEIGSIMGN
R. delemar          FFPLFRHIFIRESIQIVHGHGAFSALCHEAILH-----------NHSLFGFADTSSILTN
C. neoformans       FLPYLRHIILTENIQLVHGHGALSSLAHEAVIHAPLLRVKAVFTDHSLFGFGDAVGVLTN
A. flavus.          DTVYKQTFQPDILYVASPASLGFQILLQTRQLRKPSTVLLNYQTDLSAYSEIIFPAPLDR
A. fumigatus        KDIYKEPAQPDIVYVASPASLGFQLLLYLRQLRKPPVVLLNFQTDLSAYSEIILPSPLSR
M. circinelloides   FTRKLIKFQPDVIHLVDPVFLGAFGLAVVRYYLPNVPIVSSYHTNLAVYCDHFGFGFMTS
                                                                : : :

A. nidulans         KLLKFTLSDVDHVICVSHTCKENTVLRASLDPLMVSVIPNAVVAENFRPLHATARANERQ
F. oxysporum        KLLKFTLSDVDHSICVSHTCKENTVLRASLDPVMVSVIPNAVVAENFRPKDVPASPSPQT
S. cerevisiae       KLLTFTLTNIDRVICVSNTCKENMIVRTELSPDIISVIPNAVVSEDFKPRDPTGGTKRKQ
C. albicans         KALKFTFSDVGHVICVSHTCKENTVLRGSIDPIKVSVIPNAVISKDFKPKS---HCVNKN
R. delemar          KLLKFTLSDVDHVVCVSHTSKENTVLRAALSPKHVSVIPNAIVASRFLPDPSA-------
C. neoformans       KLLGAALRCVDEVICVSNTGRENTVLRAQLDPSIVSVIPNALEAEHFKPDPSR-------
A. flavus.          FAVWLLATVQGFLFSHPAVHTIFYPCSAVLNYLKDAGAPVERTVRLGRGVDTSLFNPTHR
A. fumigatus        WSVWLLAVVQGFLFSNPAVHTIFYPSSSILRYLKDAGAPATRAVKLGRGVDTILFHPSRR
M. circinelloides   IMWRWNRYCHSFSRFTACPSPSTMAILNDHGFEKVRLWPRGVDISLFSPLQRSESLRAQW
                              .     .                     *

A. nidulans         SGGGSQ--IQPPPRPIGPNDIITIVVISRLFYNKGTDLLIAAIPRILASHPNVRFIIAGS
F. oxysporum        TIFGSEGPVYPPPQRIGPRDTITIVVISRLFYNKGTDLLIASIPRVLENHPNTRFIIAGS
S. cerevisiae       S-----------------RDKIVIVVIGRLFPNKGSDLLTRIIPKVCSSHEDVEFIVAGD
C. albicans         Y-----------------TKEITIVVITRLFPNKGADLLTAVIPKICQLKPKVKFLIAGD
R. delemar          ----------------PDPNWITIVVISRLVYRKGIDLLVAVIPRICEAYRNVRFIIGGD
C. neoformans       ----------------ADPDWITIVVISRLVHRKGIDLLISSAPQICALFPNVRFIVGGD
A. flavus.          DN---------AYRREIAPKGEIILVCVCRLAPEKGFEFLAEATIRLAEQKIPFKLLIVGG
A. fumigatus        DE---------AFRKEIAPDGEIILVCVCRLALEKGFEFLAVAAAKLAEEKLPFKLLIVGG
M. circinelloides   MG-----------VSELKSENKTVILYVGRVSYEKNINLVIEAYKEMNHEKCHLVLVGHGP
                                           :: : *:  .*. :::     .:        ::  *

A. nidulans         GP-KAIDLEQMLERNVLQDKVEMLGAIRHEEVRDVMVRGHIYLHPSLTEAFGTVIVEAAS
F. oxysporum        GP-KAIDLEQMIETNVLQDRVEMLGPIRHEEVRDVMVRGHIYLHPSLTEAFGTVIVEAAS
S. cerevisiae       GP-KFIDFQQMIESHRLQKRVQLLGSVPHEKVRDVLCQGDIYLHASLTEAFGTILVEAAS
C. albicans         GP-KFLDLEQMREKYFLQERVTLVGAIKHEEVRDVMVQGDIYLHPSLTEAFGTVIVEAAS
R. delemar          GP-KRIDLEQMREKHGLHDRIELLGPIKHHEVRNVLIQGNIFLNTSLTEAFCIAIVEAAC
C. neoformans       GP-KMVELEQMREKYELQGRVELLGRVNPGDVRDVLTKGQIYLSNSLTEAFGISIIEAAS
A. flavus.          NRNPVVEARIHRLFDAVREHVIFTGFLTGQPLAHAYASGDIFLHCSITETFGLVVLEAMA
A. fumigatus        NRNPEVERNIHRLFDTVRDHVIFTGFLTGEPLARAYASGDLFLHCSITETFGLVVLEAMA
M. circinelloides   AF-----HEIQSYCSAKRIPVTFTGYLQGKDLSQAYASADIFAFPSVTETFGQVVLEAMS
                            .        :  : : * :    :  .   ..::   *:**:*   ::** .

A. nidulans         CGLYVVCTRVGGIPEVLPQHMTTFAK-----PEEDD--IVLATSKAISALRSNKVRTERF
F. oxysporum        CGLYVVCTQVGGIPEVLPSHMTTFAK-----PEEDD--IVLATSKAISAMRAGKIRTEKF
S. cerevisiae       CNLLIVTTQVGGIPEVLPNEMTVYAE-----QTSVSD-LVQATNKAINIIRSKALDTSSF
C. albicans         CGLYVVTTKVGGIPEVLPNEMTSFAE-----PEENS--LIDAAIDAINKIESNEIDTSKF
R. delemar          AGLFVVSTKVGGVPEVLPSHMINYAI-----PEEDD--LVIAISKAIHTFRFGKLDPSKF
C. neoformans       AGLFVVATKVGGVPEILPQDMIEFCR-----ADEDD--VIRALTHAIHTIQSSRHSPWSA
A. flavus.          SGLPVIARDQGGPSDIVQHQKTGYLV-----PPNDIR-NFVGLVRDVSINSHLRSALSTS
A. fumigatus        SGLPVVARDQGGPSDIVRHQETGYLV-----PPNDIE-TFVALVRQVSRDSQLLASLALA
M. circinelloides   SGLPVVGLDAEGVRDLVDDKRTGLLLDTFELPAKDQQKKYRDLLERLVDQPHLLGKLRRE
                    ..* ::     *  :::  .             .            :

A. nidulans         HDQVKVMYSWTDVARRTERVYKGISGDISPQEFYGYYPGEIQEAGDRVRNFALIDRLKRY
F. oxysporum        HEQVKKMYSWQNVALRTERVYDGISGTIPED---EFYGVDTSGYGSRIRNFALIDRLKRY
S. cerevisiae       HDSVSKMYDWMDVAKRTVEIYTNISSTSSADDK-------------------DWMKMVANLY
C. albicans         HDAVAKMYSWNDIARRTENVYNSLDLDKLNES--------------------LLHRLQRY
R. delemar          NNEIKDISNSTIDSSNSSGSYTTDEIKLLLLL----------------------------
C. neoformans       HTRVRDMYSWSHVSSRAEIVYLRALSRPHREIG---------------------ERMRRY
A. flavus.          ARRYAEETTWEKINNRVAWQMANAFEQRSAEESLG-------------GSD--------E
A. fumigatus        ARTYAEDTTWEKINRRVAQQMADAVEAREQAKRLQ-------------LEGQGRWCAAYE
M. circinelloides   AVKKAKTYTWYEAMECMVNVYQDAVGCSEDELPVSLKLYLKHNSDGSLVQEQELKRVNPT


A. nidulans         YGCGVWAGKLFCLCVVIDFLLYTFLEMWFPRANIDIARSWPKKLNGIDGDTAKPEKERSG
F. oxysporum        YGCGIWAGKLFCLCCVVDYLFFLFLEWWFPRDNIDICPDWPRKRPADDDASSKKGAHSTR
S. cerevisiae       KRDGIWAKHLYLLCGIVEYMLFFLLEWLYPRDEIDLAPKWPKKTVSNETKEARET-----
C. albicans         YCCGIIAGKLYALCVIVDIFIFVILEWLYPADHIDKATKWPSAIKEEDESEEETFIFPNK
R. delemar          ------------------------------------------------------------
```

```
C. neoformans       LELGPVFGIVMCCILAVEHYFFWFLEWWNPRDKIRQAINLTGAEKFEDRGKNDNK-----
A. flavus.          PVVANFMLPILEKLRLTLAVGLVYFMWLIAVVPLIIHGQRIVPRALELVHSMPVVGRCIR
A. fumigatus        KAKDRTMLALIQRIRLIAAFGFVSFMWMISVIPLIVHGSRVIPRSLAHIRGLASSRK---
M. circinelloides   ATSSSDTAENAYNEDSGDSGVEEDYALCDEESNLLLPHAEPTTMHTNKEEKKSWNNTRTY

A. nidulans         -----------
F. oxysporum        SSTSQGAPKLE
S. cerevisiae       -----------
C. albicans         VN---------
R. delemar          -----------
C. neoformans       -----------
A. flavus.          YRSR-------
A. fumigatus        -----------
M. circinelloides   LTNN-------
```

CLUSTAL 2.1 multiple sequence alignment. Sites found to confer resistance when mutated in *S. cerevisiae* are marked in red color. Sites found in the resistance-conferring state are marked in yellow color. The used individual sequences and their reference identification numbers are provided in the "Source Data" zip archive related to this manuscript.

Supplementary Methods

Compound purification methods

Purification of intermediates and final products was carried out via either normal or reverse phase chromatography. Normal phase chromatography was carried out using prepacked SiO_2 cartridges (e.g., RediSep® Rf columns from Teledyne Isco, Inc.) eluting with gradients of appropriate solvent systems (e.g., hexanes and ethyl acetate; DCM and MeOH; or unless otherwise indicated). Reverse phase chromatography was carried out using prepacked C18 cartridges (e.g., RediSep® Rf columns from Teledyne Isco, Inc.) eluting with gradients of appropriate solvent systems (e.g., acetonitrile and water; or unless otherwise indicated). Supercritical fluid chromatography (SFC purification) was carried out using the three different methods. Method 1: Princeton PPU 5 µm (100 A) column (30 x 250 mm); CO_2/MeOH. Method 2: Waters Atlantis HILIC 5µm (100 A) column (30 x 250 mm); CO_2/MeOH. Method 3: Princeton 4-EP 5µm (100 A) column (30 x 250 mm); CO_2/MeOH. Gradients were selected based on analytical separation. Reverse phase preparative HPLC purification was carried out using as follows: Phenomenex Luna C18; 5µm column (30 x 250 mm); 0.1% formic acid and 5% water in acetonitrile; 0.1% formic acid and 5% acetonitrile in water. Gradients were selected based on analytical separation.

Synthesis of compound #1

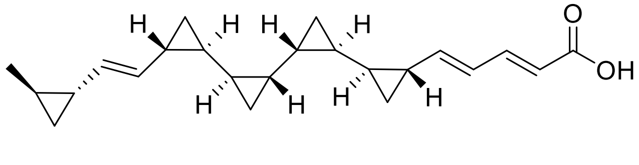

A suspension of **jawsamycin** (300 mg, 0.530 mmol, 1.0 eq) and LiOH (1270 mg, 53.0 mmol, 100 eq) in EtOH/H_2O (1:1) (6 mL) was stirred at 90 °C for 3 days. The reaction mixture was acidified with HCl conc. to pH 1-2 and extracted with DCM/H_2O. The organic extract was washed with H_2O, evaporated under reduced pressure and purified by reverse-phase flash chromatography at a gradient of 30%-100% acetonitrile in water (+0.1% formic acid) to afford **compound 1** (128 mg, 68% yield) as a white solid.

ESIMS [MH+] 339.1; Exact mass: 338.22

^{1}H NMR (400 MHz, DMSO-d_6) δ 12.02 (s, 1H), 7.07 (dd, J = 15.2, 11.1 Hz, 1H), 6.25 (dd, J = 15.0, 11.1 Hz, 1H), 5.76 (dd, J = 15.1, 9.5 Hz, 1H), 5.69 (d, J = 15.3 Hz, 1H), 5.03 – 4.91 (m, 2H), 1.31 – 1.21 (m, 1H), 1.04 – 0.94 (m, 6H), 0.76 – 0.69 (m, 1H), 0.68 – 0.47 (m, 7H), 0.46 – 0.39 (m, 1H), 0.39 – 0.28 (m, 3H), 0.16 – 0.01 (m, 4H). A ChemDraw version of the structrue of this compound is available in the “Source Data” zip archive associated with this manuscript.

Supplementary Table 1: Nucleotides

**Nu1** 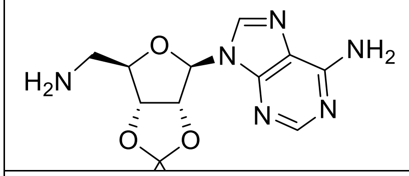	**Nu2** 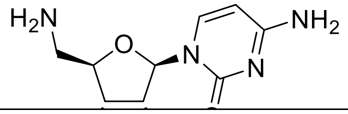	**Nu3** 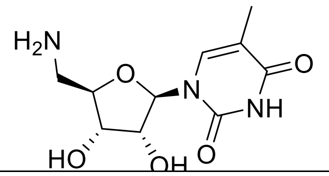
**Nu4** 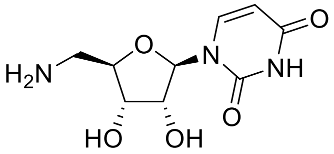	**Nu5** 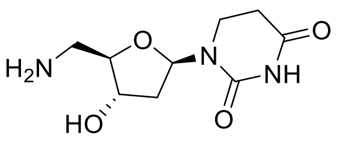	**Nu6** 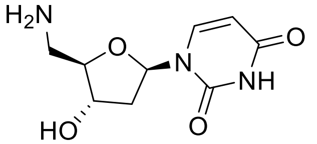
**Nu7** 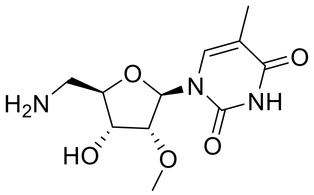	**Nu8** 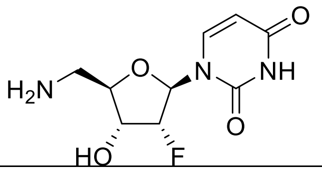	**Nu9** 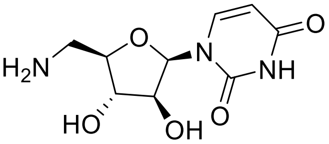
**Nu10** 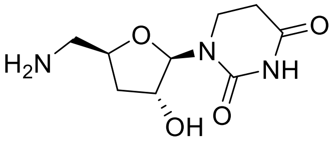	**Nu11** 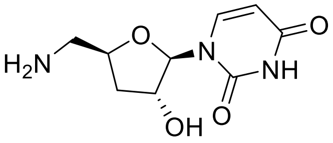	

The nucleotides in the Supplementary Table 1 are described in the literature with the exception of Nu5 and Nu10. The two nucleotides are obtained as described below for Nu5.

Synthesis of Nu5:

Step 1: A mixture of 2'-deoxyuridine (1.0g, 4.4 mmol) and Pd/C (10%)(0.20 g) in MeOH (40 mL) was stirred for 27.5 h under H_2-atmosphere (2.8 bar). The reaction mixture was filtered through a bed of celite and evaporated under reduced pressure to afford 2'-deoxy-5,6-dihydrouridine (0.99 g, 99% yield) as a grey solid.
ESIMS [MH+] 231.1; Exact mass: 230.09

Step 2: A mixture of 2'-deoxy-5,6-dihydrouridine (400 mg, 1.737 mmol, 1.0 eq), CBr_4 (691 mg, 2.085 mmol, 1.2 eq), PPh_3 (547 mg, 2.085 mmol, 1.2 eq) and NaN_3 (339 mg, 5.21 mmol, 3.0 eq) in DMF (Volume: 4 mL) was stirred at room temperature for 2 days. The reaction mixture was directly purified by normal phase column chromatography (40g) using Cyclohexane:EtOAc:MeOH as eluent (80:20:0 to 0:90:10) to afford 5'azido-2',5'-dideoxy-5,6-dihydrouridine (0.20 g, 45% yield) as a yellow oil.
ESIMS [MH+] 256.2; Exact mass: 255.10

Step 3: A suspension of 5'azido-2',5'-dideoxy-5,6-dihydrouridine (200 mg, 0.784 mmol) and Pd/C (10%) (20 mg, 9.40 µmol) in MeOH/THF (1 mL) (1:1) was stirred under H_2-atmosphere (balloon) for 1h. The reaction mixture was filtered through a bed of celite, evaporated under reduced pressure and purified by reversed phase column chromatography (13 g) using H_2O:ACN (+0.5 %TFA) as eluent (100:0 to 0:100) to afford 5'amino-2',5'-dideoxy-5,6-dihydrouridine (Nu5) (95mg, 53% yield) as beige solid.

Synthesis of compound #2 - JD-1

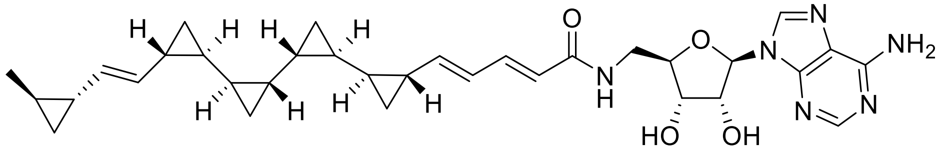

A solution of **compound 1** (15 mg, 0.044 mmol, 1.0 eq), HATU (20.22 mg, 0.053 mmol, 1.2 eq), DIEA (0.013 mL, 0.075 mmol, 1.7 eq) and **Nu1** (20.36 mg, 0.066 mmol, 1.5 eq) in DMF (0.5 mL) was stirred at room temperature for 1 h. The reaction mixture was extracted with EtOAc/ H_2O. The org. extract was evaporated under reduced pressure to yield a crude product that was used without further purification in the deprotection-step.
A solution of the crude product (27.6 mg, 0.044 mmol) in H_2O/THF/TFA (3mL) (1:1:1) was stirred at room temperature for 3 days. The reaction mixture was quenched with $NaHCO_3$ (aq. sat) and extracted with DCM. The org. extract was evaporated under reduced pressure and purified by SFC-purification (method 2) to afford **compound 2** (5.3 mg, 19% yield) as a white solid**.**
ESIMS [MH+] 587.5; Exact mass: 586.33
^{1}H NMR (400 MHz, DMSO-d_6) δ: 8.38 (s, 1H), 8.26 (t, J = 6.0 Hz, 1H), 8.22 (s, 1H), 7.57 (s, 2H), 6.96 (dd, J = 14.9, 11.1 Hz, 1H), 6.21 (dd, J = 15.0, 11.1 Hz, 1H), 5.95 – 5.82 (m, 2H), 5.65 (dd, J = 15.0, 9.4 Hz, 1H), 5.04 – 4.89 (m, 2H), 4.71 – 4.61 (m, 1H), 4.09 – 4.03 (m, 1H), 4.00 – 3.93 (m, 1H), 3.51 – 3.44 (m, 2H), 1.30 – 1.21 (m, 1H), 1.04 – 0.93 (m, 6H), 0.76 – 0.69 (m, 1H), 0.69 – 0.47 (m, 7H), 0.46 – 0.40 (m, 1H), 0.39 – 0.28 (m, 3H), 0.15 – -0.00 (m, 4H). A ChemDraw version of the structrue of this compound is available in the "Source Data" zip archive associated with this manuscript.

Synthesis of compound #3 - JD-2

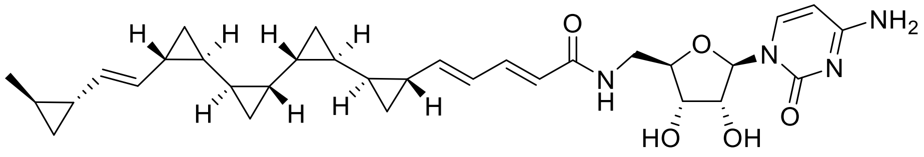

A solution of **compound 1** (30 mg, 0.089 mmol, 1.0 eq), **Nu2** (21.47 mg, 0.089 mmol, 1.0 eq), HATU (40.4 mg, 0.106 mmol, 1.2 eq) and DIEA (0.026 mL, 0.151 mmol, 1.7 eq) in DMF (0.5 mL) was stirred at room temperature for 1 h. The reaction mixture was extracted with EtOAc/H_2O. The org. extract was evaporated under reduced pressure and purified by SFC-purification (method 2) to afford **compound 3** (9.2 mg, 17% yield) as a white solid.

ESIMS [MH+] 563.4; Exact mass: 562.32

^{1}H NMR (400 MHz, DMSO-*d*$_6$) δ: 8.12 (t, J = 6.0 Hz, 1H), 7.60 (d, J = 7.5 Hz, 1H), 7.32 – 7.09 (m, 2H), 6.94 (dd, J = 15.1, 11.2 Hz, 1H), 6.19 (dd, J = 15.0, 11.1 Hz, 1H), 5.89 (d, J = 15.0 Hz, 1H), 5.76 – 5.60 (m, 3H), 5.28 (d, J = 5.7 Hz, 1H), 5.08 (d, J = 4.8 Hz, 1H), 5.00 – 4.92 (m, 2H), 4.03 – 3.97 (m, 1H), 3.86 – 3.77 (m, 2H), 3.57 – 3.21 (m, 2H), 1.29 – 1.20 (m, 1H), 1.04 – 0.93 (m, 6H), 0.76 – 0.69 (m, 1H), 0.68 – 0.47 (m, 7H), 0.45 – 0.39 (m, 1H), 0.37 – 0.27 (m, 3H), 0.14 – 0.00 (m, 4H). A ChemDraw version of the structrue of this compound is available in the "Source Data" zip archive associated with this manuscript.

Synthesis of compound #4 - JD-3

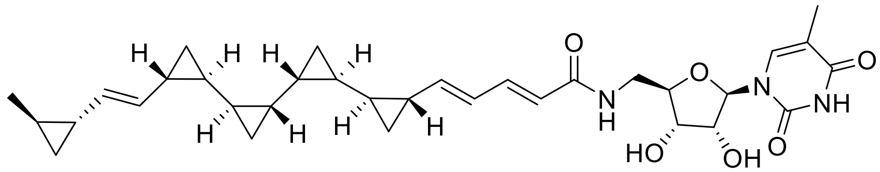

A solution of **compound 1** (15 mg, 0.044 mmol, 1.0 eq), **Nu3** (28.9 mg, 0.044 mmol, 1.0 eq), HATU (20.22 mg, 0.053 mmol, 1.2 eq) and DIEA (0.023 mL, 0.133 mmol, 1.7 eq) in DMF (0.5 mL) was stirred at room temperature for 30 min. The reaction mixture was extracted with EtOAc/brine. The org. extract was washed with H_2O, dried with $MgSO_4$, filtered and evaporated under reduced pressure. The crude product was suspended in MeCN and sonicated for 5min. The suspension was filtered to afford **compound 4** (15 mg, 57% yield) as a white solid.

ESIMS [MH+] 578.5; Exact mass: 577.32

^{1}H NMR (400 MHz, DMSO-*d*$_6$) δ 11.31 (s, 1H), 8.09 (t, *J* = 6.0 Hz, 1H), 7.47 (s, 1H), 6.95 (dd, *J* = 15.0, 11.1 Hz, 1H), 6.20 (dd, *J* = 15.0, 11.2 Hz, 1H), 5.88 (d, *J* = 15.1 Hz, 1H), 5.74 (d, *J* = 6.0 Hz, 1H), 5.64 (dd, *J* = 15.0, 9.4 Hz, 1H), 5.33 (d, *J* = 5.8 Hz, 1H), 5.13 (d, *J* = 5.1 Hz, 1H), 5.03 – 4.91 (m, 2H), 4.08 – 3.99 (m, 1H), 3.91 – 3.84 (m, 1H), 3.83 – 3.75 (m, 1H), 3.54 – 3.44 (m, 1H), 3.30 – 3.26 (m, 1H), 1.79 (s, 3H), 1.31 – 1.13 (m, 1H), 1.04 – 0.93 (m, 6H), 0.76 – 0.47 (m, 8H), 0.46 – 0.39 (m, 1H), 0.38 – 0.28 (m, 3H), 0.15 – 0.01 (m, 4H). A ChemDraw version of the structrue of this compound is available in the "Source Data" zip archive associated with this manuscript.

Synthesis of compound #5 - JD-4

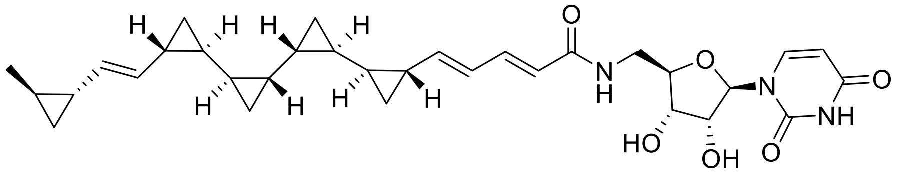

A solution of **compound 1** (10 mg, 0.030 mmol, 1.0 eq), **Nu4** (7.19 mg, 0.030 mmol, 1.0 eq), HATU (13.48 mg, 0.035 mmol, 1.2 eq) and DIEA (7.74 µl, 0.044 mmol, 1.5 eq) in DMF (0.5 mL) was stirred at room temperature for 1 h. The reaction mixture was extracted with EtOAc/brine. The org. extract was evaporated under reduced pressure and purified by SFC-purification (method 2) to afford **compound 5** (7.4 mg, 42% yield) as a white solid**.**

ESIMS [MH+] 564.3; Exact mass: 563.30

^{1}H NMR (400 MHz, DMSO-d_6) δ 11.34 (s, 1H), 8.08 (t, *J* = 6.0 Hz, 1H), 7.67 (d, *J* = 8.1 Hz, 1H), 6.94 (dd, *J* = 15.0, 11.1 Hz, 1H), 6.20 (dd, *J* = 15.0, 11.2 Hz, 1H), 5.88 (d, *J* = 15.1 Hz, 1H), 5.72 (d, *J* = 5.7 Hz, 1H), 5.68 – 5.58 (m, 2H), 5.37 (d, *J* = 5.7 Hz, 1H), 5.15 (d, *J* = 5.1 Hz, 1H), 5.02 – 4.91 (m, 2H), 4.12 – 3.99 (m, 1H), 3.90 – 3.76 (m, 2H), 3.52 – 3.42 (m, 1H), 3.35 – 3.22 (m, 1H), 1.31 – 1.20 (m, 1H), 1.05 – 0.91 (m, 6H), 0.76 – 0.69 (m, 1H), 0.68 – 0.47 (m, 7H), 0.46 – 0.39 (m, 1H), 0.38 – 0.26 (m, 3H), 0.15 – -0.00 (m, 4H). A ChemDraw version of the structrue of this compound is available in the “Source Data” zip archive associated with this manuscript..

Synthesis of compound #6 - JD-5

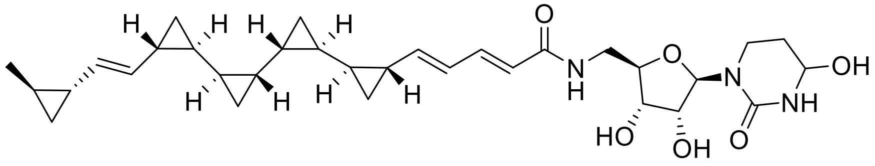

A suspension of **jawsamycin** (50 mg, 0.088 mmol, 1.0 eq) and NaBH4 (66.9 mg, 1.769 mmol, 20.0 eq) in MeOH was stirred at room temperature for 1h. The reaction mixture was extracted with DCM/H_2O. The org. extract was evaporated under reduced pressure and purified by normal phase column chromatography (4 g silica) using Cyclohexane:EtOAc:MeOH as eluent (50:50:0 to 0:80:20), followed by a purification by reverse-phase HPLC (method 1) to afford **compound 6** (9.2 mg, 17% yield, diastereomeric mixture) as a white solid.

ESIMS [MH+] 568.5; Exact mass: 567.33

^{1}H NMR (400 MHz, DMSO-d_6) δ: 8.03 (t, J = 5.9 Hz, 1H), 7.02 (dd, J = 25.5, 4.1 Hz, 1H), 6.93 (dd, J = 15.0, 11.1 Hz, 1H), 6.18 (dd, J = 15.2, 11.2, 1.4 Hz, 1H), 5.89 (d, J = 15.0 Hz, 1H), 5.75 – 5.57 (m, 2H), 5.57 – 5.50 (m, 1H), 4.99 – 4.88 (m, 4H), 4.81 – 4.73 (m, 1H), 3.95 – 3.84 (m, 1H), 3.73 – 3.65 (m, 1H), 3.65 – 3.56 (m, 1H), 3.46 – 3.26 (m, 2H), 3.26 – 3.06 (m, 2H), 1.84 – 1.57 (m, 2H), 1.32 – 1.18 (m, 1H), 0.98 (t, J = 5.0 Hz, 6H), 0.79 – 0.68 (m, 1H), 0.69 – 0.47 (m, 7H), 0.46 – 0.40 (m, 1H), 0.39 – 0.27 (m, 3H), 0.16 – -0.02 (m, 4H). A ChemDraw version of the structrue of this compound is available in the “Source Data” zip archive associated with this manuscript.

Synthesis of compound #7 - JD-6

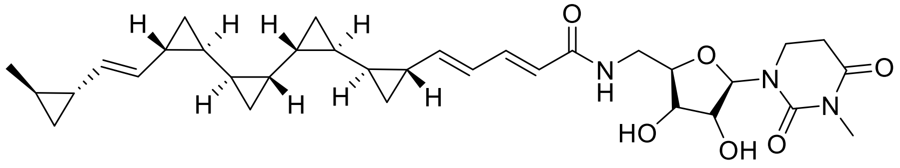

A solution of **jawsamycin** (20 mg, 0.035 mmol, 1.0 eq), K_2CO_3 (9.77 mg, 0.071 mmol, 2.0 eq) and CH_3I (6.02 mg, 0.042 mmol, 1.2 eq) in DMF (0.5 mL) was stirred at room temperature for 2 h. The reaction mixture was extracted with EtOAc/H_2O. The org. extract was evaporated under reduced pressure and purified by SFC-purification (method 1) to afford **compound 7** (3.2 mg, 14% yield) as a white solid.

ESIMS [MH+] 580.5; Exact mass: 579.33

^{1}H NMR (400 MHz, DMSO-d_6) δ 8.00 (t, *J* = 6.0 Hz, 1H), 6.93 (dd, *J* = 15.0, 11.1 Hz, 1H), 6.19 (dd, *J* = 15.0, 11.2 Hz, 1H), 5.88 (d, *J* = 15.0 Hz, 1H), 5.72 – 5.58 (m, 2H), 5.13 (d, *J* = 5.7 Hz, 1H), 5.04 (d, *J* = 5.0 Hz, 1H), 4.99 – 4.95 (m, 2H), 3.98 – 3.91 (m, 1H), 3.78 – 3.72 (m, 1H), 3.72 – 3.67 (m, 1H), 3.43 – 3.14 (m, 4H), 2.99 (s, 3H), 2.71 – 2.54 (m, 2H), 1.30 – 1.21 (m, 1H), 1.05 – 0.93 (m, 6H), 0.76 – 0.68 (m, 1H), 0.68 – 0.47 (m, 7H), 0.45 – 0.39 (m, 1H), 0.38 – 0.28 (m, 3H), 0.15 – 0.01 (m, 4H). A ChemDraw version of the structrue of this compound is available in the "Source Data" zip archive associated with this manuscript.

Synthesis of compound #8 - JD-7

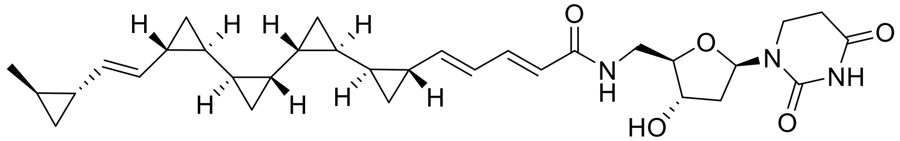

A solution of **compound 1** (15 mg, 0.044 mmol, 1.0 eq), **Nu5** (23.00 mg, 0.053 mmol, 1.0 eq), HATU (20.2 mg, 0.053 mmol, 1.2 eq) and DIEA (0.012 mL, 0.066 mmol, 1.5 eq) in DMF (0.5 mL) was stirred at room temperature for 1 h. The reaction mixture was extracted with EtOAc/brine. The org. extract was evaporated under reduced pressure and purified by SFC-purification (method 2) to afford **compound 8** (6.6 mg, 26% yield) as a white solid.

ESIMS [MH+] 550.5; Exact mass: 549.32

^{1}H NMR (400 MHz, DMSO-d_6) δ: 10.24 (s, 1H), 8.01 (t, J = 6.0 Hz, 1H), 6.95 (dd, J = 15.0, 11.2 Hz, 1H), 6.21 (dd, J = 15.1, 11.1 Hz, 1H), 6.11 (t, J = 7.2 Hz, 1H), 5.89 (d, J = 15.1 Hz, 1H), 5.65 (dd, J = 15.0, 9.5 Hz, 1H), 5.22 – 5.17 (m, 1H), 5.02 – 4.94 (m, 2H), 4.09 – 3.97 (m, 1H), 3.69 – 3.48 (m, 1H), 3.46 – 3.11 (m, 4H), 2.59 – 2.44 (m, 2H), 2.13 – 2.01 (m, 1H), 1.90 – 1.77 (m, 1H), 1.32 – 1.19 (m, 1H), 1.07 – 0.94 (m, 6H), 0.78 – 0.71 (m, 1H), 0.70 – 0.48 (m, 7H), 0.47 – 0.41 (m, 1H), 0.41 – 0.29 (m, 3H), 0.16 – -0.02 (m, 4H). A ChemDraw version of the structrue of this compound is available in the "Source Data" zip archive associated with this manuscript.

Synthesis of compound #9 - JD-8

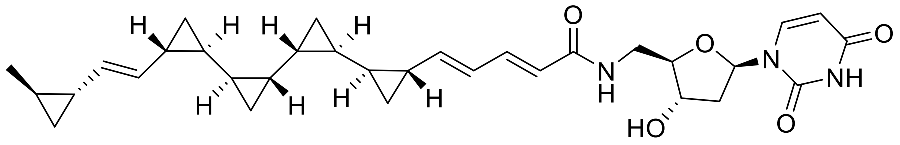

A solution of **compound 1** (20 mg, 0.059 mmol, 1.0 eq), **Nu6** (13.43 mg, 0.059 mmol, 1.0 eq), HATU (27.0 mg, 0.071 mmol, 1.2 eq) and DIEA (0.015 mL, 0.089 mmol, 1.5 eq) in DMF (0.5 mL) was stirred at room temperature for 30 min**.** The reaction mixture was extracted with EtOAc/brine. The org. extract evaporated under reduced pressure and purified by SFC-purification (method 3) to afford **compound 9** (8.2 mg, 24% yield) as a white solid

ESIMS [MH+] 548.4; Exact mass: 547.30

^{1}H NMR (400 MHz, DMSO-d_6) δ: 11.31 (s, 1H), 8.07 (t, J = 5.9 Hz, 1H), 7.66 (d, J = 8.2 Hz, 1H), 6.94 (dd, J = 15.0, 11.1 Hz, 1H), 6.20 (dd, J = 15.0, 11.2 Hz, 1H), 6.11 (t, J = 6.9 Hz, 1H), 5.87 (d, J = 15.0 Hz, 1H), 5.72 – 5.54 (m, 2H), 5.30 (d, J = 4.2 Hz, 1H), 5.03 – 4.88 (m, 2H), 4.18 – 4.08 (m, 1H), 3.81 – 3.72 (m, 1H), 3.48 – 3.38 (m, 1H), 3.36 – 3.20 (m, 1H), 2.15 – 2.03 (m, 2H), 1.31 – 1.20 (m, 1H), 1.05 – 0.91 (m, 6H), 0.76 – 0.69 (m, 1H), 0.69 – 0.47 (m, 7H), 0.46 – 0.40 (m, 1H), 0.38 – 0.28 (m, 3H), 0.15 – -0.00 (m, 4H). A ChemDraw version of the structrue of this compound is available in the “Source Data” zip archive associated with this manuscript.

Synthesis of compound #10 - JD-9

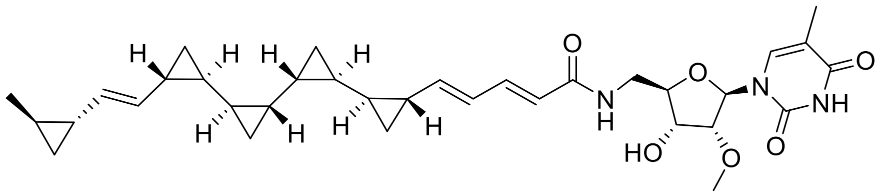

A solution of **compound 1** (15 mg, 0.044 mmol, 1,0 eq), **Nu7** (12.02 mg, 0.044 mmol, 1.0 eq), HATU (20.22 mg, 0.053 mmol, 1.2 eq) and DIEA (0.012 mL, 0.066 mmol, 1.5 eq) in DMF (0.5 mL) was stirred at room temperature for 1 h.

The reaction mixture was extracted with EtOAc/brine. The org. extract was evaporated under reduced pressure and purified by SFC-purification (method 2) to afford **compound 10** (12.6 mg, 45% yield) as a white solid.

ESIMS [MH+] 592.5; Exact mass: 591.33

^{1}H NMR (400 MHz, DMSO-d_6) δ: 11.35 (s, 1H), 8.10 (t, J = 6.0 Hz, 1H), 7.48 (s, 1H), 6.95 (dd, J = 15.0, 11.1 Hz, 1H), 6.20 (dd, J = 15.0, 11.2 Hz, 1H), 5.88 (d, J = 15.1 Hz, 1H), 5.82 (d, J = 5.6 Hz, 1H), 5.64 (dd, J = 15.0, 9.4 Hz, 1H), 5.22 (d, J = 5.9 Hz, 1H), 5.02 – 4.91 (m, 2H), 4.07 – 3.98 (m, 1H), 3.87 – 3.76 (m, 2H), 3.56 – 3.45 (m, 1H), 3.39 – 3.23 (m, 4H), 1.79 (s, 3H), 1.33 – 1.22 (m, 1H), 1.06 – 0.92 (m, 6H), 0.78 – 0.69 (m, 1H), 0.69 – 0.47 (m, 7H), 0.46 – 0.39 (m, 1H), 0.39 – 0.27 (m, 3H), 0.15 – -0.00 (m, 4H). A ChemDraw version of the structrue of this compound is available in the “Source Data” zip archive associated with this manuscript.

Synthesis of compound #11 - JD-10

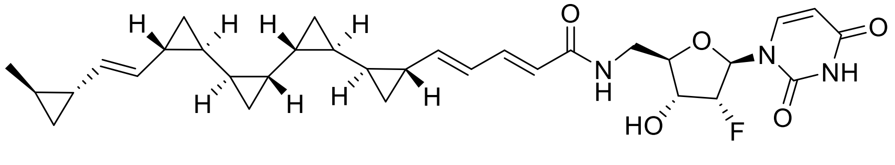

A solution of **compound 1** (25 mg, 0.074 mmol, 1.0 eq), **Nu8** (25.8 mg, 0.089 mmol, 1.2 eq), HATU (33.7 mg, 0.089 mmol, 1.2 eq) and DIEA (0.022 mL, 0.126 mmol, 1.5 eq) in DMF (0.5 mL) was stirred at room temperature for 30 min.
The reaction mixture was extracted with EtOAc/H_2O. The org. extract was evaporated under reduced pressure and purified by SFC-purification (method 1) to afford **compound 11** (21 mg, 47% yield) as a white solid.
ESIMS [MH+] 566.4; Exact mass: 565.30
^{1}H NMR (400 MHz, DMSO-d_6) δ: 11.40 (s, 1H), 8.13 (t, J = 5.9 Hz, 1H), 7.65 (d, J = 8.1 Hz, 1H), 6.94 (dd, J = 15.0, 11.2 Hz, 1H), 6.20 (dd, J = 15.0, 11.1 Hz, 1H), 5.91 – 5.76 (m, 2H), 5.71 – 5.56 (m, 3H), 5.22 – 5.03 (m, 1H), 5.03 – 4.92 (m, 2H), 4.14 – 3.97 (m, 1H), 3.90 – 3.80 (m, 1H), 3.68 – 3.56 (m, 1H), 3.37 – 3.23 (m, 1H), 1.31 – 1.20 (m, 1H), 1.06 – 0.92 (m, 6H), 0.76 – 0.69 (m, 1H), 0.68 – 0.47 (m, 7H), 0.46 – 0.40 (m, 1H), 0.38 – 0.27 (m, 3H), 0.15 – 0.01 (m, 4H). A ChemDraw version of the structrue of this compound is available in the "Source Data" zip archive associated with this manuscript.

Synthesis of compound #12 - JD-11

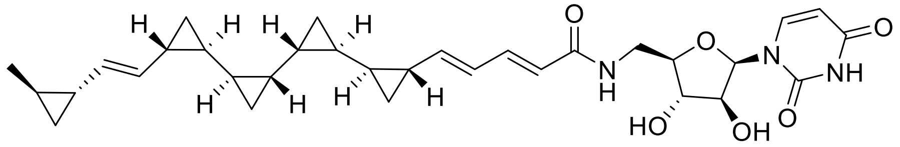

A solution of **compound 1** (35 mg, 0.103 mmol, 1.0 eq), **Nu9** (25.1 mg, 0.103 mmol, 1.0 eq), HATU (47.2 mg, 0.124 mmol, 1.2 eq) and DIEA (0.031 mL, 0.176 mmol, 1.7 eq) in DMF (0.5 mL) was stirred at room temperature for 1 h. The reaction mixture was extracted with EtOAc/H_2O. The org. extract was evaporated under reduced pressure and purified by SFC-purification (method 2) to afford **compound 12** (28 mg, 46% yield) as a white solid.
ESIMS [MH+] 564.4; Exact mass: 563.30
^{1}H NMR (400 MHz, DMSO-d_6) δ 11.26 (s, 1H), 8.10 (t, *J* = 6.0 Hz, 1H), 7.63 (d, *J* = 8.1 Hz, 1H), 6.94 (dd, *J* = 15.0, 11.1 Hz, 1H), 6.20 (dd, *J* = 15.0, 11.1 Hz, 1H), 5.97 (d, *J* = 3.7 Hz, 1H), 5.86 (d, *J* = 15.0 Hz, 1H), 5.69 – 5.60 (m, 2H), 5.54 – 5.47 (m, 2H), 5.02 – 4.92 (m, 2H), 3.97 – 3.92 (m, 1H), 3.87 – 3.83 (m, 1H), 3.83 – 3.77 (m, 1H), 3.49 – 3.36 (m, 2H), 1.30 – 1.20 (m, 1H), 1.05 – 0.93 (m, 6H), 0.77 – 0.69 (m, 1H), 0.68 – 0.47 (m, 7H), 0.46 – 0.39 (m, 1H), 0.39 – 0.29 (m, 3H), 0.15 – 0.01 (m, 4H). A ChemDraw version of the structrue of this compound is available in the "Source Data" zip archive associated with this manuscript.

Synthesis of compound #13 - JD-12

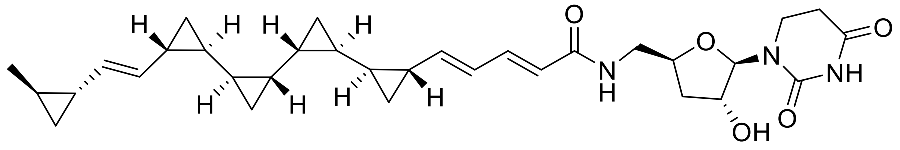

A solution of **compound 1** (30 mg, 0.089 mmol, 1.0 eq), **Nu10** (24.38 mg, 0.106 mmol, 1.2 eq), HATU (40.4 mg, 0.106 mmol, 1.2 eq) and DIEA (0.026 mL, 0.151 mmol, 1.7 eq) in DMF (0.5 mL) was stirred at room temperature for 1 h. The reaction mixture was extracted with EtOAc/H_2O. The org. extract was evaporated under reduced pressure and purified by SFC-purification (method 3) to afford **compound 13** (27 mg, 53% yield) as a white solid.

ESIMS [MH+] 550.4; Exact mass: 549.32

^{1}H NMR (400 MHz, DMSO-d_6) δ 10.23 (s, 1H), 8.03 (t, *J* = 5.9 Hz, 1H), 6.93 (dd, *J* = 15.0, 11.2 Hz, 1H), 6.19 (dd, *J* = 15.0, 11.2 Hz, 1H), 5.88 (d, *J* = 15.1 Hz, 1H), 5.68 – 5.57 (m, 2H), 5.28 (d, *J* = 4.3 Hz, 1H), 5.03 – 4.90 (m, 2H), 4.20 – 4.13 (m, 1H), 4.12 – 4.03 (m, 1H), 3.43 – 3.19 (m, 4H), 2.61 – 2.41 (m, 2H), 1.85 – 1.72 (m, 2H), 1.30 – 1.20 (m, 1H), 1.06 – 0.92 (m, 6H), 0.77 – 0.69 (m, 1H), 0.69 – 0.47 (m, 7H), 0.46 – 0.39 (m, 1H), 0.39 – 0.28 (m, 3H), 0.16 – 0.00 (m, 4H). A ChemDraw version of the structrue of this compound is available in the "Source Data" zip archive associated with this manuscript.

Synthesis of compound #14 - JD-13

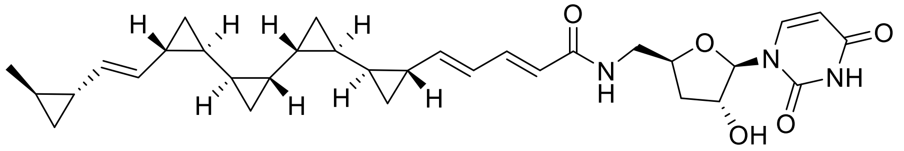

A solution of **compound 1** (20 mg, 0.059 mmol, 1.0 eq), **Nu11** (16.11 mg, 0.071 mmol, 1.2 eq), HATU (27.0 mg, 0.071 mmol, 1.2 eq) and DIEA (0.018 mL, 0.100 mmol, 1.5 eq) in DMF (0.5 mL) was stirred at room temperature for 30 min.
The reaction mixture was extracted with EtOAc/H_2O. The org. extract was evaporated under reduced pressure and purified by SFC-purification (method 2) to afford **compound 14** (4.8 mg, 14% yield) as a white solid.

ESIMS [MH+] 548.5; Exact mass: 547.30

^{1}H NMR (400 MHz, DMSO-d_6) δ 11.30 (s, 1H), 8.13 (t, *J* = 6.0 Hz, 1H), 7.60 (d, *J* = 8.1 Hz, 1H), 6.94 (dd, *J* = 15.0, 11.1 Hz, 1H), 6.20 (dd, *J* = 15.0, 11.2 Hz, 1H), 5.88 (d, *J* = 15.0 Hz, 1H), 5.69 – 5.50 (m, 4H), 5.02 – 4.91 (m, 2H), 4.29 – 4.20 (m, 2H), 3.49 – 3.25 (m, 2H), 1.89 – 1.75 (m, 2H), 1.30 – 1.21 (m, 1H), 1.05 – 0.92 (m, 6H), 0.76 – 0.69 (m, 1H), 0.69 – 0.47 (m, 7H), 0.46 – 0.40 (m, 1H), 0.39 – 0.29 (m, 3H), 0.14 – 0.01 (m, 4H). A ChemDraw version of the structrue of this compound is available in the "Source Data" zip archive associated with this manuscript.

Synthesis of compound #15 - JD-14

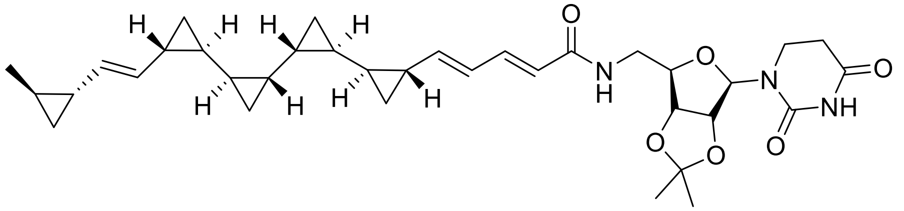

A suspension of **jawsamycin** (1.0g, 1.768 mmol, 1.0 eq) and pTsOH (3.36 g, 17.68 mmol, 10 eq) in acetone (200 mL) was stirred at room temperature for 1.5 h. The reaction mixture was quenched with $NaHCO_3$ (1N) and extracted with EtOAc. The organic extract was evaporated under reduced pressure and purified by normal phase column chromatography (40g silica) using Cyclohexane:

EtOAc as eluent (100:0 to 0:100) to afford **compound 15** (870 mg, 74% yield) as a pale yellow solid. A ChemDraw version of the structrue of this compound is available in the "Source Data" zip archive associated with this manuscript.

ESIMS [MH+] 606.4; Exact mass: 605.35

^{1}H NMR (400 MHz, DMSO-d_6) δ: 10.34 (s, 1H), 8.06 (t, J = 6.0 Hz, 1H), 6.94 (dd, J = 15.1, 11.1 Hz, 1H), 6.20 (dd, J = 15.0, 11.1 Hz, 1H), 5.86 (d, J = 15.1 Hz, 1H), 5.72 – 5.60 (m, 2H), 5.01 – 4.92 (m, 2H), 4.86 (dd, J = 6.6, 2.9 Hz, 1H), 4.54 (dd, J = 6.7, 4.7 Hz, 1H), 3.93 – 3.80 (m, 1H), 3.45 – 3.26 (m, 4H), 2.65 – 2.56 (m, 1H), 2.56 – 2.45 (m, 1H), 1.45 (s, 3H), 1.31 – 1.20 (m, 4H), 1.04 – 0.92 (m, 6H), 0.77 – 0.69 (m, 1H), 0.68 – 0.46 (m, 7H), 0.45 – 0.39 (m, 1H), 0.38 – 0.28 (m, 3H), 0.15 – -0.00 (m, 4H). A ChemDraw version of the structrue of this compound is available in the "Source Data" zip archive associated with this manuscript.

Synthesis of compound #16

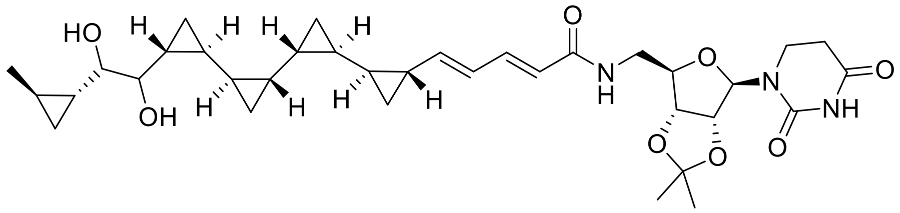

A solution of **compound 15** (870 mg, 1.436 mmol, 1.0 eq)**,** methanesulfonamide (546 mg, 5.74 mmol, 4.0 eq) and AD-mix-alpha (2702 mg, 5.74 mmol, 4.0 eq) in tert. Butanol/H_2O (1:1) (40 mL) was stirred at room temperature for 25 h. The reaction mixture was quenched with NaS_2O_3 (1M) and extracted with EtOAc. The organic extract was evaporated under reduced pressure and purified by normal phase column chromatography (40 g silica) using DCM: MeOH as eluent (100:0 to 80:20) to afford **compound 16** (605 mg, 66% yield) as a diastereomeric mixture.

ESIMS [MH+] 640.5; Exact mass: 639.35.

Synthesis of compound #17

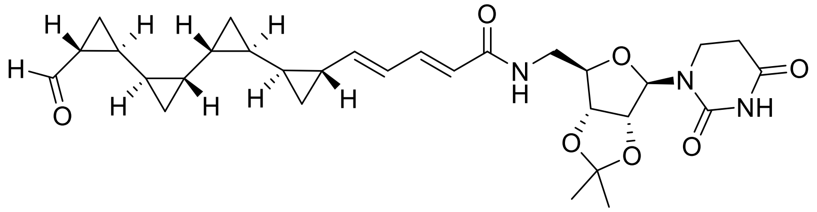

A solution of **compound 16** (355 mg, 0.555 mmol, 1.0 eq) and diacetoxyiodobenzene (214 mg, 0.666 mmol, 1.2 eq) in DCM (4 mL) was stirred at room temperature for 30min. The reaction mixture was evaporated under reduced pressure and purified by normal phase column chromatography (24 g silica) using DCM:MeOH as eluent (99:01 to 90:10) to afford **compound 17** (180mg, 32% yield) as a white solid.

ESIMS [MH+] 554.3; Exact mass: 553.28

^{1}H NMR (400 MHz, DMSO-d_6) δ: 10.35 (s, 1H), 8.86 (d, J = 5.7 Hz, 1H), 8.07 (t, J = 6.0 Hz, 1H), 6.95 (dd, J = 15.0, 11.1 Hz, 1H), 6.21 (dd, J = 15.0, 11.2 Hz, 1H), 5.87 (d, J = 15.0 Hz, 1H), 5.74 – 5.59 (m, 2H), 4.87 (dd, J = 6.7, 2.9 Hz, 1H), 4.55 (dd, J = 6.7, 4.6 Hz, 1H), 3.90 – 3.82 (m, 1H), 3.46 – 3.28 (m, 4H), 2.70 – 2.44 (m, 2H), 1.63 – 1.55 (m, 1H), 1.54 – 1.42 (m, 4H), 1.33 – 1.22 (m, 4H), 1.20 – 1.12 (m, 1H), 1.03 – 0.95 (m, 1H), 0.93 – 0.85 (m, 1H), 0.71 – 0.54 (m, 6H), 0.26 – 0.19 (m, 1H), 0.18 – 0.07 (m, 3H).

Synthesis of compound #18

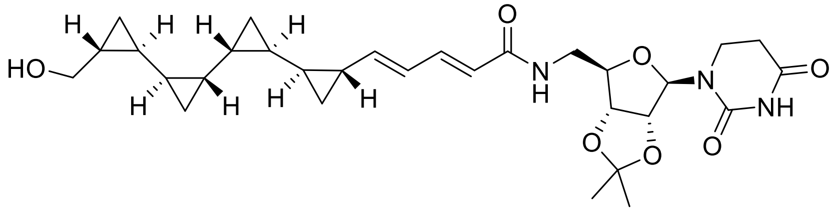

A solution of **compound 17** (50 mg, 0.090 mmol, 1.0 eq) and $NaBH_4$ (4.10 mg, 0.108 mmol, 1.2 eq) in MeOH (2 mL) was stirred at room temperature for 15 min. The reaction mixture was quenched with NH_4Cl (aq. 1N) and extracted with DCM. The organic extract was evaporated under reduced pressure and purified by normal phase column chromatography (4 g silica) using DCM:MeOH as eluent (100:0 to 80:20) to afford **compound 18** (24 mg, 42% yield) as a white solid.
ESIMS [MH+] 556.3; Exact mass: 555.29.

Synthesis of compound #19 - JD-17

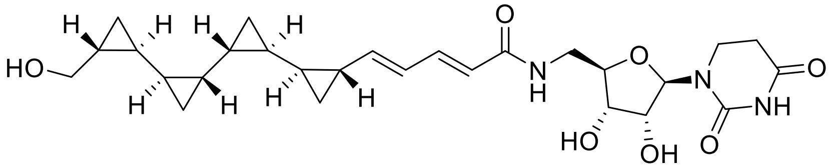

A solution of **compound 18** (24 mg, 0.043 mmol) and TFA (0.2 mL) in H_2O/THF (10:1) (5mL), was stirred at room temperature for 18 h. The reaction mixture was quenched with $NaHCO_3$ (aq. sat) and extracted with DCM. The organic extract was evaporated under reduced pressure and purified by SFC chromatography (method 2) to afford **compound 19** (3.1 mg, 13% yield) as a beige solid.
ESIMS [MH+] 516.3; Exact mass: 515.26
^{1}H NMR (400 MHz, DMSO-d_6) δ: 10.26 (s, 1H), 8.01 (t, J = 5.9 Hz, 1H), 6.93 (dd, J = 15.0, 11.2 Hz, 1H), 6.19 (dd, J = 15.0, 11.1 Hz, 1H), 5.88 (d, J = 15.0 Hz, 1H), 5.70 – 5.58 (m, 2H), 5.12 (d, J = 5.8 Hz, 1H), 5.01 (d, J = 5.1 Hz, 1H), 4.35 (t, J = 5.6 Hz, 1H), 3.96 – 3.89 (m, 1H), 3.77 – 3.72 (m, 1H), 3.71 – 3.65 (m, 1H), 3.43 – 3.09 (m, 6H), 2.60 – 2.39 (m, 2H), 1.31 – 1.19 (m, 1H), 1.02 – 0.93 (m, 1H), 0.68 – 0.43 (m, 8H), 0.21 – 0.01 (m, 6H). A ChemDraw version of the structrue of this compound is available in the "Source Data" zip archive associated with this manuscript.

Synthesis of compound #20

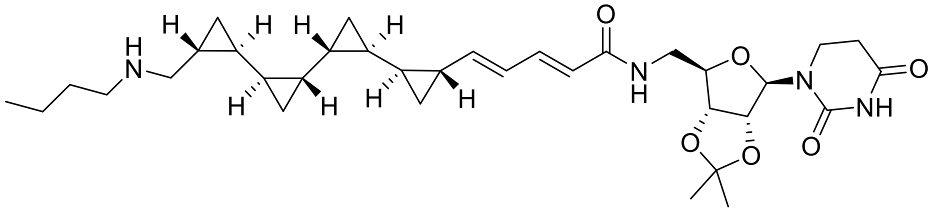

A solution of **compound 17** (50 mg, 0.090 mmol, 1.0 eq), n-butylamine (7.93 mg, 0.108 mmol, 1.2 eq) and $NaBH(OAc)_3$ (22.97 mg, 0.108 mmol, 1.2 eq) in DCE (1 mL) was stirred at room temperature for 30 min. The reaction mixture was quenched with $NaHCO_3$

(aq. sat) and extracted with DCM. The organic extract was evaporated under reduced pressure and purified by normal phase column chromatography (12 g silica) using DCM:MeOH as eluent (100:0 to 80:20) to afford **compound 20** (26 mg, 34% yield) as a yellow oil.
ESIMS [MH+] 611.4; Exact mass: 610.37

Synthesis of compound #21 - JD-18

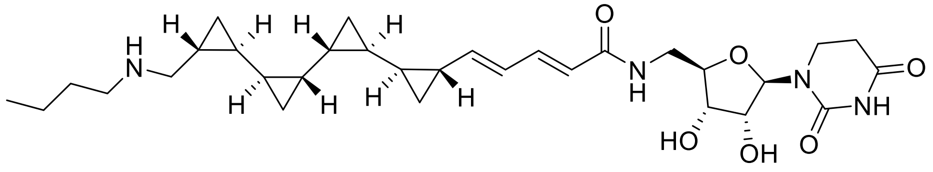

A solution of **compound 20** (26 mg, 0.043 mmol) and TFA (0.05 mL) in H_2O/THF (2:1) (0.6mL) was stirred at room temperature for 18 h. The reaction mixture was directly purified by reverse-phase HPLC (method 1) to afford **compound 21** (2.6 mg, 9% yield) as a white solid.
ESIMS [MH+] 571.3; Exact mass: 570.34
^{1}H NMR (400 MHz, DMSO-d_6) δ: 10.26 (s, 1H), 8.02 (t, J = 5.9 Hz, 1H), 6.93 (dd, J = 15.0, 11.1 Hz, 1H), 6.19 (dd, J = 15.0, 11.1 Hz, 1H), 5.88 (d, J = 15.0 Hz, 1H), 5.70 – 5.58 (m, 2H), 3.98 – 3.90 (m, 1H), 3.79 – 3.72 (m, 1H), 3.71 – 3.64 (m, 1H), 3.46 – 3.13 (m, 4H), 2.83 – 2.69 (m, 3H), 2.62 – 2.42 (m, 3H), 1.57 – 1.46 (m, 2H), 1.38 – 1.19 (m, 3H), 0.97 (tt, J = 7.5, 4.3 Hz, 1H), 0.89 (t, J = 7.3 Hz, 3H), 0.80 – 0.63 (m, 2H), 0.62 – 0.48 (m, 6H), 0.38 – 0.24 (m, 2H), 0.18 – 0.02 (m, 4H). A ChemDraw version of the structrue of this compound is available in the "Source Data" zip archive associated with this manuscript.

Synthesis of compound #22

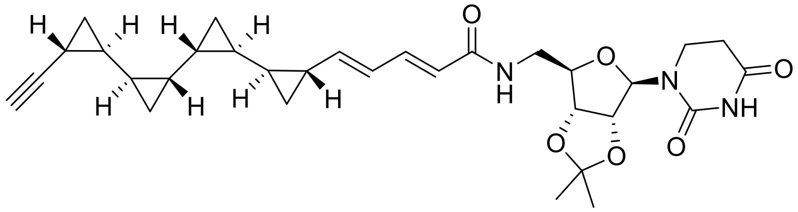

A solution of **compound 17** (60 mg, 0.108 mmol, 1.0 eq), dimethyl (1-diazo-2-oxopropyl)phosphonate (104 mg, 0.542 mmol, 5.0 eq) and K_2CO_3 (105 mg, 0.759 mmol, 7.0 eq) in MeOH/THF (1:1) (2mL) was stirred at room temperature for 5 h. The reaction mixture was extracted with EtOAc/H_2O. The organic extract was evaporated under reduced pressure and purified by reverse-phase HPLC (method 1) to afford **compound 22** (48 mg, 77% yield) as a colourless resin.
ESIMS [MH+] 550.3; Exact mass: 549.28

Synthesis of compound #23 - JD-19

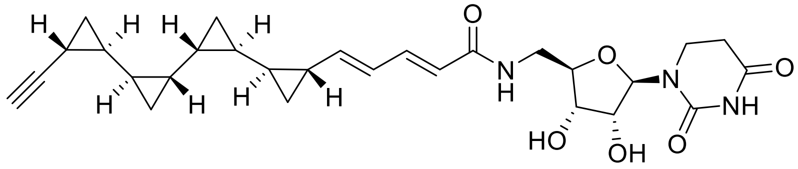

A solution of **compound 22** (48 mg, 0.087 mmol) and TFA (0.1 mL) in H_2O/THF (2:1) (0.6mL) was stirred at room temperature for 4 h. The reaction mixture was directly purified by reverse-phase HPLC (method 1) to afford **compound 23** (8 mg, 17% yield) as a white solid.

ESIMS [MH+] 510.1; Exact mass: 509.25

^{1}H NMR (400 MHz, DMSO-d_6) δ: 10.25 (s, 1H), 8.00 (t, J = 5.9 Hz, 1H), 6.93 (dd, J = 15.0, 11.1 Hz, 1H), 6.19 (dd, J = 15.0, 11.1 Hz, 1H), 5.88 (d, J = 15.0 Hz, 1H), 5.70 – 5.57 (m, 2H), 5.11 (d, J = 5.7 Hz, 1H), 5.01 (d, J = 5.1 Hz, 1H), 3.98 – 3.89 (m, 1H), 3.78 – 3.72 (m, 1H), 3.71 – 3.64 (m, 1H), 3.44 – 3.13 (m, 4H), 2.59 – 2.43 (m, 3H), 1.30 – 1.20 (m, 1H), 1.10 – 1.02 (m, 1H), 1.02 – 0.93 (m, 2H), 0.67 – 0.48 (m, 8H), 0.17 – 0.03 (m, 4H). A ChemDraw version of the structrue of this compound is available in the "Source Data" zip archive associated with this manuscript.

Synthesis of compound #24

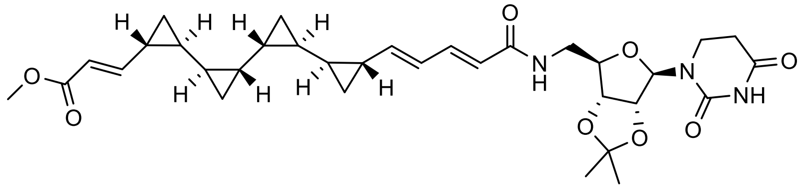

A solution of **compound 17** (60 mg, 0.108 mmol, 1.0 eq) and methyl 2-(triphenylphosphoranylidene)acetate (36.2 mg, 0.108 mmol, 1.0 eq) was stirred in DCM (1.5 mL) at room temperature for 4 h. The reaction was diluted with H_2O and was extracted with DCM. The organic extract was evaporated under reduced pressure and purified by reverse-phase HPLC (method 1) to afford **compound 24** (27 mg, 39% yield) as a white solid.

ESIMS [MH+] 610.2; Exact mass: 609.31

^{1}H NMR (400 MHz, DMSO-d_6) δ: 10.34 (s, 1H), 8.06 (t, J = 6.0 Hz, 1H), 6.94 (dd, J = 15.0, 11.1 Hz, 1H), 6.41 (dd, J = 15.4, 10.1 Hz, 1H), 6.20 (dd, J = 15.0, 11.2 Hz, 1H), 5.93 – 5.82 (m, 2H), 5.73 – 5.60 (m, 2H), 4.86 (dd, J = 6.7, 2.9 Hz, 1H), 4.54 (dd, J = 6.7, 4.6 Hz, 1H), 3.91 – 3.81 (m, 1H), 3.60 (s, 3H), 3.44 – 3.18 (m, 4H), 2.70 – 2.43 (m, 2H), 1.45 (s, 3H), 1.43 – 1.34 (m, 1H), 1.32 – 1.20 (m, 4H), 1.20 – 1.10 (m, 1H), 1.02 – 0.93 (m, 1H), 0.78 – 0.66 (m, 2H), 0.65 – 0.51 (m, 6H), 0.19 – 0.00 (m, 4H).

Synthesis of compound #25 - JD-20

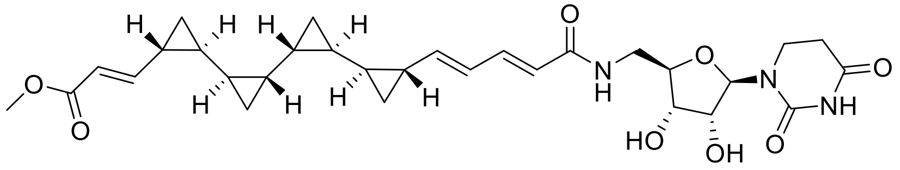

To a solution of **compound 24** (27 mg, 0.044 mmol) in H_2O/THF (2:1) (0.6mL) was added TFA (0.1mL). The reaction mixture was stirred at room temperature for 4 h. The reaction mixture was directly purified by reverse-phase HPLC (method 1) to afford **compound 25** as a white solid (4.5 mg, 17% yield) as a white solid.

ESIMS [MH+] 570.2; Exact mass: 569.27

^{1}H NMR (400 MHz, DMSO-d_6) δ: 10.25 (s, 1H), 8.00 (t, J = 5.9 Hz, 1H), 6.93 (dd, J = 15.0, 11.1 Hz, 1H), 6.41 (dd, J = 15.4, 10.1 Hz, 1H), 6.19 (dd, J = 15.0, 11.1 Hz, 1H), 5.93 – 5.82 (m, 2H), 5.70 – 5.57 (m, 2H), 5.13 (d, J = 5.5 Hz, 1H), 5.03 (d, J = 5.0 Hz, 1H), 3.99 – 3.89

(m, 1H), 3.77 – 3.72 (m, 1H), 3.71 – 3.65 (m, 1H), 3.60 (s, 3H), 3.46 – 3.15 (m, 4H), 2.60 – 2.43 (m, 2H), 1.43 – 1.33 (m, 1H), 1.31 – 1.20 (m, 1H), 1.19 – 1.08 (m, 1H), 1.02 – 0.94 (m, 1H), 0.78 – 0.66 (m, 2H), 0.64 – 0.52 (m, 6H), 0.17 – 0.03 (m, 4H). A ChemDraw version of the structrue of this compound is available in the "Source Data" zip archive associated with this manuscript.
