# Supplementary material for: Jawsamycin exhibits in vivo antifungal properties by inhibiting Spt14/Gpi3-mediated biosynthesis of glycosylphosphatidylinositol
Source: Nat Commun. 2020 Jul 7;11:3387. doi: 10.1038/s41467-020-17221-5 (PMC7341893; doi:10.1038/s41467-020-17221-5)
Supplement: Supplementary file 3 — Reporting Summary [file 41467_2020_17221_MOESM3_ESM.pdf]

nature research

Corresponding author(s): Dominic Hoepfner
Jianshi Tao

Last updated by author(s): May 14, 2020

Reporting Summary

Nature Research wishes to improve the reproducibility of the work that we publish. This form provides structure for consistency and transparency in reporting. For further information on Nature Research policies, see Authors & Referees and the Editorial Policy Checklist .

Statistics

For all statistical analyses, confirm that the following items are present in the figure legend, table legend, main text, or Methods section.

n/a	Confirmed	
☐	☒	The exact sample size (*n*) for each experimental group/condition, given as a discrete number and unit of measurement
☐	☒	A statement on whether measurements were taken from distinct samples or whether the same sample was measured repeatedly
☐	☒	The statistical test(s) used AND whether they are one- or two-sided<br>*Only common tests should be described solely by name; describe more complex techniques in the Methods section.*
☒	☐	A description of all covariates tested
☒	☐	A description of any assumptions or corrections, such as tests of normality and adjustment for multiple comparisons
☐	☒	A full description of the statistical parameters including central tendency (e.g. means) or other basic estimates (e.g. regression coefficient) AND variation (e.g. standard deviation) or associated estimates of uncertainty (e.g. confidence intervals)
☐	☒	For null hypothesis testing, the test statistic (e.g. *F*, *t*, *r*) with confidence intervals, effect sizes, degrees of freedom and *P* value noted<br>*Give P values as exact values whenever suitable.*
☒	☐	For Bayesian analysis, information on the choice of priors and Markov chain Monte Carlo settings
☒	☐	For hierarchical and complex designs, identification of the appropriate level for tests and full reporting of outcomes
☒	☐	Estimates of effect sizes (e.g. Cohen's *d*, Pearson's *r*), indicating how they were calculated

Our web collection on statistics for biologists contains articles on many of the points above.

Software and code

Policy information about availability of computer code

Data collection	Compound screen: Hamamatsu FDSS7000 Software<br>HIP HOP Microarray: Affymetrix, GeneTitan Software<br>Yeast growth curves: Beckman Coulter DTX880 Plate reader with Multimode Detection Software v3.3.09<br>Flow cytometry: BD FACSaria Fusion with FACSDiva 8.0.1
Data analysis	TIBCO Spotfire 7.9 -10.3<br>Microsoft Excel 16.3 for Mac<br>Graphpad Prism 6 - 8.3<br>FlowJo 10.1r1<br>Helios (internal package see DOI: 10.1177/2472555217752140)

For manuscripts utilizing custom algorithms or software that are central to the research but not yet described in published literature, software must be made available to editors/reviewers. We strongly encourage code deposition in a community repository (e.g. GitHub). See the Nature Research guidelines for submitting code & software for further information.

Data

Policy information about availability of data

All manuscripts must include a data availability statement. This statement should provide the following information, where applicable:

- Accession codes, unique identifiers, or web links for publicly available datasets
- A list of figures that have associated raw data
- A description of any restrictions on data availability

Raw data has been provided as requested and organization is described in the "Data Availability" section.

Field-specific reporting

Please select the one below that is the best fit for your research. If you are not sure, read the appropriate sections before making your selection.

- [x] Life sciences
- [ ] Behavioural & social sciences
- [ ] Ecological, evolutionary & environmental sciences

For a reference copy of the document with all sections, see nature.com/documents/nr-reporting-summary-flat.pdf

Life sciences study design

All studies must disclose on these points even when the disclosure is negative.

Sample size	For survival studies, 10 mice/group would provide at least 80% power to test the hazard ratio of 0.2 or more with a level of significance p= 0.025 using Log Rank test and Cox proportional model (one sided test) assuming 100% and 50% mortality in the control and treated groups, respectively. For the tissue pathogen burden, 10 mice/group would provide at least 90% power to detect the effect size of 3 or 3 SD difference in CFU (expressed as log) by two sample t-test with α of 0.05, assuming the standard deviation of the treated group is twice of the one for the control group. A two sample t-test and ANOVA were used with the post-hoc analysis using Tukey correction methods to control for the overall type error rate of 0.05. For all comparisons, median (Interquartile range), and 95% confidence interval were computed. All data analyses were conducted using GraphPad Prism 6. p< 0.05 were considered significant.
Data exclusions	No data was excluded apart from 77 of 14720 wells in the primary screen that were automatically masked for quality control reasons
Replication	As outlined in the manuscript, almost every conclusion derived from data was validated by orthogonal or follow-up assays: e.g. pooled HIP HOP data was validated by single strain growth curves, identified resistance-confirming mutations were introduced into a naïve wild-type background and re-tested, etc. Number of replicates performed are indicated in the corresponding figure legends. All assays are well established in-house are regularly used for internal programs and have been deployed to generate data for similar publications reporting compounds with antifungal properties (e.g. doi: 10.1016/j.chembiol.2017.12.007, doi: 10.1016/j.micres.2013.11.004 or doi: 10.1128/AAC.01809-12). All deployed assays are robust and no sequential repeats were necessary to obtain the data published in this study.
Randomization	Randomization has been done for the mouse experiment. Animals were infected and put unlabeled in one cage prior to treatment. Only once the first treatment was done they were transferred into labelled cages.
Blinding	The person from the biological research facility that checked the mice twice daily and reported the status did not know the study protocol nor the drug under investigation.

Reporting for specific materials, systems and methods

We require information from authors about some types of materials, experimental systems and methods used in many studies. Here, indicate whether each material, system or method listed is relevant to your study. If you are not sure if a list item applies to your research, read the appropriate section before selecting a response.

Materials & experimental systems

n/a	Involved in the study	
x		Antibodies
	x	Eukaryotic cell lines
x		Palaeontology
	x	Animals and other organisms
x		Human research participants
x		Clinical data

Methods

n/a	Involved in the study	
x		ChIP-seq
	x	Flow cytometry
x		MRI-based neuroimaging

Eukaryotic cell lines

Policy information about cell lines

Cell line source(s)	HCT116 cells (CCL-247), HEK293 (CRL11268), K562 (CCL-243), and HEPG2 (HB-8065) cell lines and microbial strains listed in Table 1 where obtained from ATCC. Yeast strains were obtained from OpenBiosystems (YSC1056 and YSC1055).
Authentication	Microbial strains were verified by morphology (spores, hyphae, yeast) the used mammalian cell lines are periodically verified by an external servide provider using the Promega STR system.
Mycoplasma contamination	Used mammalian cell lines (HCT116 cells, HEK293, K562, and HEPG2 are routinely tested for mycoplasma contamination using the Lonza MycoAlert mycoplasma detection kit. All results were negative.
Commonly misidentified lines (See ICLAC register)	None of the lines listed in the ICLAC register v.10 was used

Animals and other organisms

Policy information about studies involving animals; ARRIVE guidelines recommended for reporting animal research

Laboratory animals	Mus musculus, ICR mice, purchased from Taconic (Germantown, NY) were used. Experiments were conducted using male CD1 outbred mice weighing 25-30 gm. Mice were housed in animal care systems vented caging with a floor area of 75 square inches (484 square centimeters). The ambient temperature was maintained between 68-79 F, humidity 30-70% and a 12h dark/light cycle.
Wild animals	The study does not use wild animals
Field-collected samples	The study does not contain field-collected samples
Ethics oversight	All animal related study procedures were compliant with the Animal Welfare Act, the Guide for the Care and Use of Laboratory Animals, and the Office of Laboratory Animal Welfare and were conducted under an IACUC approved protocol by the Lundquist Institute at Harbor-UCLA Medical Center.

Note that full information on the approval of the study protocol must also be provided in the manuscript.

Flow Cytometry

Plots

Confirm that:

- [x] The axis labels state the marker and fluorochrome used (e.g. CD4-FITC).
- [x] The axis scales are clearly visible. Include numbers along axes only for bottom left plot of group (a 'group' is an analysis of identical markers).
- [x] All plots are contour plots with outliers or pseudocolor plots.
- [x] A numerical value for number of cells or percentage (with statistics) is provided.

Methodology

Sample preparation	After 4 days treatment with jawsamycin or DMSO, the cells were split and stained using a 1:100 dilution of the FLAER reagent (FLAER Alexa Fluor 488 proaerolysin, FL2S, Cedarlanelabs) in PBS + 3%BSA for 20 min.
Instrument	BD FACSAria Fusion
Software	BD FACSDiva 8.0.1, FlowJo 10.1r1
Cell population abundance	At least 10'000 cells were analyzed per condition. As observed effects were robust (> or < 90%) this was judged sufficient.
Gating strategy	As a single cell type was analysed gating strategy was straightforward by using positive and negative controls: for jawsamycin effect testing, untreated and unstained cells were used to set the gates for negative events. Untreated, FLAER-stained cells were used to set the gates for positive events. In the genetic control experiment, FLAER-stained cells with the non-targeting sgRNA were used to set gates for positive events and unstained cells were used as a negative control.

- [x] Tick this box to confirm that a figure exemplifying the gating strategy is provided in the Supplementary Information.
