# Supplementary material for: Jawsamycin exhibits in vivo antifungal properties by inhibiting Spt14/Gpi3-mediated biosynthesis of glycosylphosphatidylinositol
Source: Nat Commun. 2020 Jul 7;11:3387. doi: 10.1038/s41467-020-17221-5 (PMC7341893; doi:10.1038/s41467-020-17221-5)
Supplement: Supplementary file 4 — Source Data [file 41467_2020_17221_MOESM4_ESM.zip › Source Data/SourceData_FungalSPT14Sequences.docx]

**Fungal SPT14/GPI3 seqences used for multiple sequence alingment (Supplemental Figure 3)**

>SPT14/ YPL175W Saccharomyces cerevisiae

MGFNIAMLCDFFYPQLGGVEFHIYHLSQKLIDLGHSVVIITHAYKDRVGVRHLTNGLKVYHVPFFVIFRE

TTFPTVFSTFPIIRNILLREQIQIVHSHGSASTFAHEGILHANTMGLRTVFTDHSLYGFNNLTSIWVNKL

LTFTLTNIDRVICVSNTCKENMIVRTELSPDIISVIPNAVVSEDFKPRDPTGGTKRKQSRDKIVIVVIGR

LFPNKGSDLLTRIIPKVCSSHEDVEFIVAGDGPKFIDFQQMIESHRLQKRVQLLGSVPHEKVRDVLCQGD

IYLHASLTEAFGTILVEAASCNLLIVTTQVGGIPEVLPNEMTVYAEQTSVSDLVQATNKAINIIRSKALD

TSSFHDSVSKMYDWMDVAKRTVEIYTNISSTSSADDKDWMKMVANLYKRDGIWAKHLYLLCGIVEYMLFF

LLEWLYPRDEIDLAPKWPKKTVSNETKEARET

>CBF89087.1 Aspergillus nidulans

MPYNIAMVSDFFFPQPGGVESHIYQLSTKLIDRGHKVIIITHAYKGRTGVRYLTNGLKVYHVPFLVIYRE

TTMPTVFSFFPIFRNIVIREQIQIVHGHQSLSSFCHEAILHARTMGLRTAFTDHSLFGFADAGSILTNKL

LKFTLSDVDHVICVSHTCKENTVLRASLDPLMVSVIPNAVVAENFRPLHATARANERQSGGGSQIQPPPR

PIGPNDIITIVVISRLFYNKGTDLLIAAIPRILASHPNVRFIIAGSGPKAIDLEQMLERNVLQDKVEMLG

AIRHEEVRDVMVRGHIYLHPSLTEAFGTVIVEAASCGLYVVCTRVGGIPEVLPQHMTTFAKPEEDDIVLA

TSKAISALRSNKVRTERFHDQVKVMYSWTDVARRTERVYKGISGDISPQEFYGYYPGEIQEAGDRVRNFA

LIDRLKRYYGCGVWAGKLFCLCVVIDFLLYTFLEMWFPRANIDIARSWPKKLNGIDGDTAKPEKERSG

>XP_002373141.1 Aspergillus flavus

MASFTSKPDDFPKILKGRRVLLTTESLGPVNGVSRTTLSLVEYLRRNGVDLAVVAPQYQGFRYQAQDAAD

CRIPGYPLPYNPDLTIVYPFRLDTVYKQTFQPDILYVASPASLGFQILLQTRQLRKPSTVLLNYQTDLSA

YSEIIFPAPLDRFAVWLLATVQGFLFSHPAVHTIFYPCSAVLNYLKDAGAPVERTVRLGRGVDTSLFNPT

HRDNAYRREIAPKGEIILVCVCRLAPEKGFEFLAEATIRLAEQKIPFKLLIVGGNRNPVVEARIHRLFDA

VREHVIFTGFLTGQPLAHAYASGDIFLHCSITETFGLVVLEAMASGLPVIARDQGGPSDIVQHQKTGYLV

PPNDIRNFVGLVRDVSINSHLRSALSTSARRYAEETTWEKINNRVAWQMANAFEQRSAEESLGGSDEPVV

ANFMLPILEKLRLTLAVGLVYFMWLIAVVPLIIHGQRIVPRALELVHSMPVVGRCIRYRSR

>XP_753071.1 Aspergillus fumigatus

MDRESFPQSLKGKKVLLTTESLGPVNGVSRTTGSLIDYLRRNEVDLMVVAPKFAGAQQAEQPEANLRLPG

YPLPYNPDLTLVYPFRLKDIYKEPAQPDIVYVASPASLGFQLLLYLRQLRKPPVVLLNFQTDLSAYSEII

LPSPLSRWSVWLLAVVQGFLFSNPAVHTIFYPSSSILRYLKDAGAPATRAVKLGRGVDTILFHPSRRDEA

FRKEIAPDGEIILVCVCRLALEKGFEFLAVAAAKLAEEKLPFKLLIVGGNRNPEVERNIHRLFDTVRDHV

IFTGFLTGEPLARAYASGDLFLHCSITETFGLVVLEAMASGLPVVARDQGGPSDIVRHQETGYLVPPNDI

ETFVALVRQVSRDSQLLASLALAARTYAEDTTWEKINRRVAQQMADAVEAREQAKRLQLEGQGRWCAAYE

KAKDRTMLALIQRIRLIAAFGFVSFMWMISVIPLIVHGSRVIPRSLAHIRGLASSRK

>EIE83077.1 Rhizopus delemar

MGGVESHLYELSQRLIQRGHKVIIVTHAYGNRTGVRYLTNGLKVYYVPAKVIYSEATLPTIYGFFPLFRH

IFIRESIQIVHGHGAFSALCHEAILHNHSLFGFADTSSILTNKLLKFTLSDVDHVVCVSHTSKENTVLRA

ALSPKHVSVIPNAIVASRFLPDPSAPDPNWITIVVISRLVYRKGIDLLVAVIPRICEAYRNVRFIIGGDG

PKRIDLEQMREKHGLHDRIELLGPIKHHEVRNVLIQGNIFLNTSLTEAFCIAIVEAACAGLFVVSTKVGG

VPEVLPSHMINYAIPEEDDLVIAISKAIHTFRFGKLDPSKFNNEIKDISNSTIDSSNSSGSYTTDEIKLL

LLL

>FOXG_00935 Fusarium oxysporum

MGLRTVFTDHSLFGFADAGTILTNKLLKFTLSDVDHSICVSHTCKENTVLRASLDPVMVSVIPNAVVAEN

FRPKDVPASPSPQTTIFGSEGPVYPPPQRIGPRDTITIVVISRLFYNKGTDLLIASIPRVLENHPNTRFI

IAGSGPKAIDLEQMIETNVLQDRVEMLGPIRHEEVRDVMVRGHIYLHPSLTEAFGTVIVEAASCGLYVVC

TQVGGIPEVLPSHMTTFAKPEEDDIVLATSKAISAMRAGKIRTEKFHEQVKKMYSWQNVALRTERVYDGI

SGTIPEDEFYGVDTSGYGSRIRNFALIDRLKRYYGCGIWAGKLFCLCCVVDYLFFLFLEWWFPRDNIDIC

PDWPRKRPADDDASSKKGAHSTRSSTSQGAPKLE

>EPB84151.1 Mucor circinelloides

MRIAIITENFLPKVDGVTRTLARLLEHLSKMGHQVLLLGPETNMTTYAGANLVGTYGIPFFLYPELKFNF

WRPKFTRKLIKFQPDVIHLVDPVFLGAFGLAVVRYYLPNVPIVSSYHTNLAVYCDHFGFGFMTSIMWRWN

RYCHSFSRFTACPSPSTMAILNDHGFEKVRLWPRGVDISLFSPLQRSESLRAQWMGVSELKSENKTVILY

VGRVSYEKNINLVIEAYKEMNHEKCHLVLVGHGPAFHEIQSYCSAKRIPVTFTGYLQGKDLSQAYASADI

FAFPSVTETFGQVVLEAMSSGLPVVGLDAEGVRDLVDDKRTGLLLDTFELPAKDQQKKYRDLLERLVDQP

HLLGKLRREAVKKAKTYTWYEAMECMVNVYQDAVGCSEDELPVSLKLYLKHNSDGSLVQEQELKRVNPTA

TSSSDTAENAYNEDSGDSGVEEDYALCDEESNLLLPHAEPTTMHTNKEEKKSWNNTRTYLTNN

>XP_717350.1 Candida albicans

MGYNIAMVTDFFYPQPGGVEFHVYHLSQKLIELGHSVVIITHNYSSRNGVRVLTNGLKVYYVPLWVIYRS

SVFPTVFSCFPILRNIFIRENIEIIHGHGSFSTLCHEAILHGRTMGLKTVFTDHSLFGFAEIGSIMGNKA

LKFTFSDVGHVICVSHTCKENTVLRGSIDPIKVSVIPNAVISKDFKPKSHCVNKNYTKEITIVVITRLFP

NKGADLLTAVIPKICQLKPKVKFLIAGDGPKFLDLEQMREKYFLQERVTLVGAIKHEEVRDVMVQGDIYL

HPSLTEAFGTVIVEAASCGLYVVTTKVGGIPEVLPNEMTSFAEPEENSLIDAAIDAINKIESNEIDTSKF

HDAVAKMYSWNDIARRTENVYNSLDLDKLNESLLHRLQRYYCCGIIAGKLYALCVIVDIFIFVILEWLYP

ADHIDKATKWPSAIKEEDESEEETFIFPNKVN

>AFR95039.2 Cryptococcus neoformans

MWKVIYYLNLALYTVLLLTTSFIAVLIAIVCSLTGRRLNTNYFVARTFYHFAGPILGWKFQVEGEQYLWE

LSGEHGGGKAGEKGRSMVMVGNHQSFVDILYLGRIFPKHAAIMAKKSIQWIPGLGWFMMMSGTVFINRSN

NKSAIASLQHAGEEMKRKRISLWIFPEGTRHNTPEPELLNFKKGAFYLAVQAGVPIVPVVCENYNHLFNG

KSHFRRGTLRIKVLPPIPTTGLTTADVPNLIEKTRNAMLETLREISTPSQSTSQAGSPDPLLGRPGRERE

NYYTSGSPAPPEGVSSAAEIGAEEEAEAAVEDAIGREEADNGERHVHVVGKNDRGDETMSSPKRLAIAMV

SDFFFPVIGGVEGHIYSLSVELMRRGHKVIVITHSHPDRLGIHYLGPSLKVYYLPYLPIASSASLPNFLL

FLPYLRHIILTENIQLVHGHGALSSLAHEAVIHAPLLRVKAVFTDHSLFGFGDAVGVLTNKLLGAALRCV

DEVICVSNTGRENTVLRAQLDPSIVSVIPNALEAEHFKPDPSRADPDWITIVVISRLVHRKGIDLLISSA

PQICALFPNVRFIVGGDGPKMVELEQMREKYELQGRVELLGRVNPGDVRDVLTKGQIYLSNSLTEAFGIS

IIEAASAGLFVVATKVGGVPEILPQDMIEFCRADEDDVIRALTHAIHTIQSSRHSPWSAHTRVRDMYSWS

HVSSRAEIVYLRALSRPHREIGERMRRYLELGPVFGIVMCCILAVEHYFFWFLEWWNPRDKIRQAINLTG

AEKFEDRGKNDNK
