# Supplementary figures and images for: Jawsamycin exhibits in vivo antifungal properties by inhibiting Spt14/Gpi3-mediated biosynthesis of glycosylphosphatidylinositol
Source: Nat Commun. 2020 Jul 7;11:3387. doi: 10.1038/s41467-020-17221-5 (PMC7341893; doi:10.1038/s41467-020-17221-5)

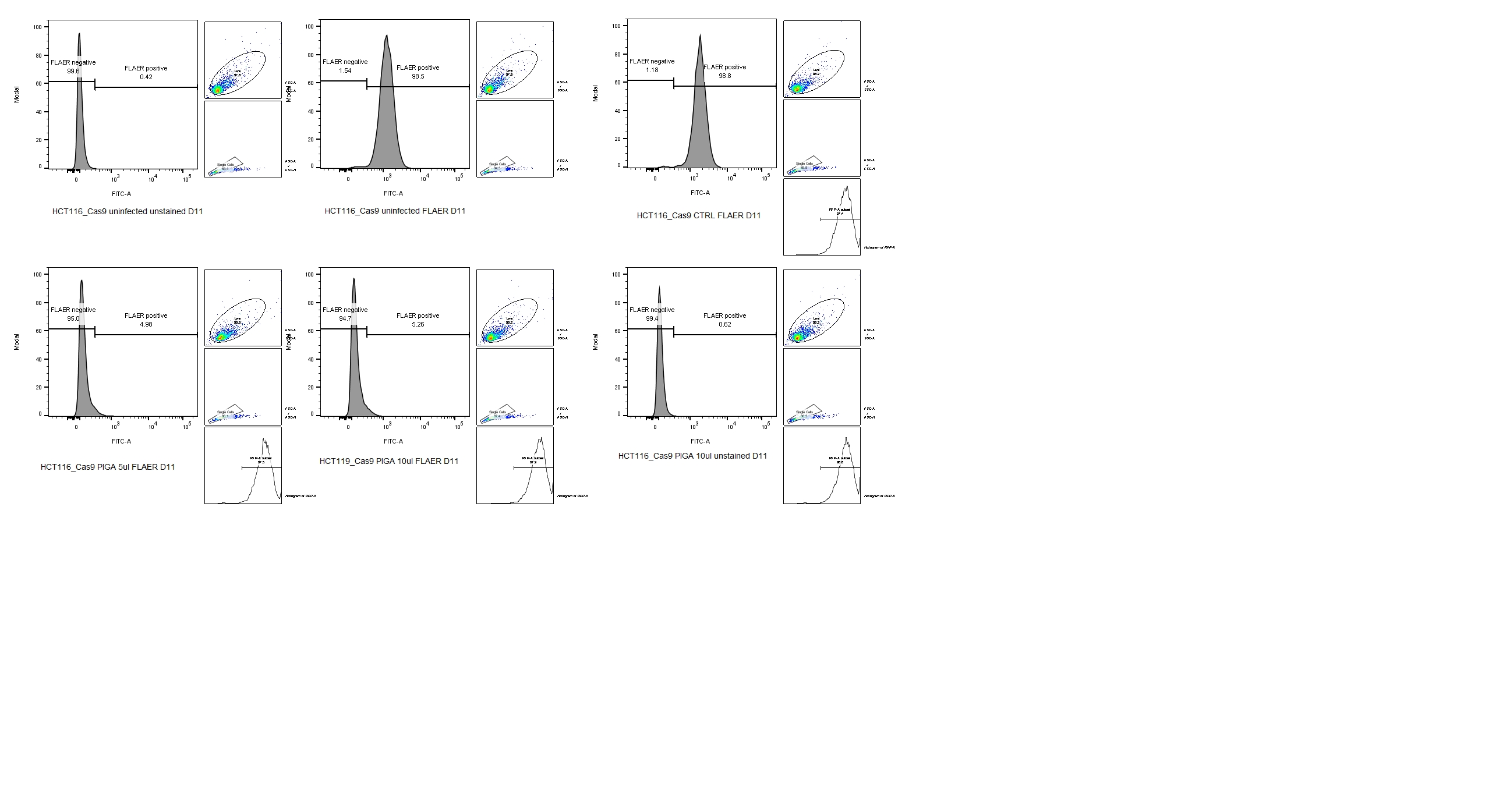

Supplement: Supplementary file 4 — Source Data [file 41467_2020_17221_MOESM4_ESM.zip › Source Data/SourceData_FACS/Figure 3b/190909_HCT116_FLAER_D11-Layout_modal.jpg]

# BD FACSDiva 8.0.1

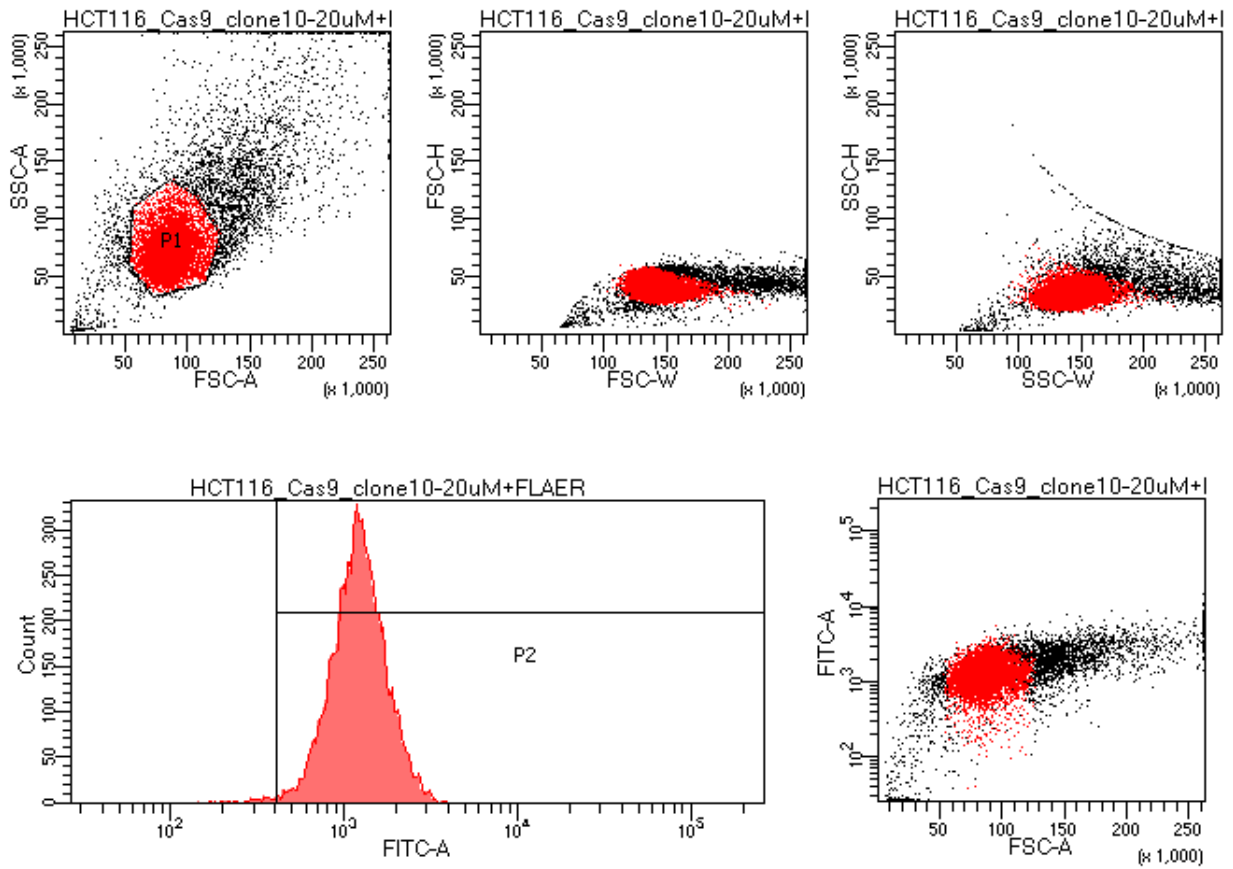

| Tube: 20uM+FLAER |         |         |        |
|------------------|---------|---------|--------|
| Population       | #Events | %Parent | %Total |
| ■ All Events     | 10,000  | ####    | 100.0  |
| ■ P1             | 7,143   | 71.4    | 71.4   |
| ▣ P2             | 7,008   | 98.1    | 70.1   |

Supplement: Supplementary file 4 — Source Data [file 41467_2020_17221_MOESM4_ESM.zip › Source Data/SourceData_FACS/Figure 3c/HCT116_Cas9_clone10_20uM+FLAER_01032016103802.pdf]

# BD FACSDiva 8.0.1

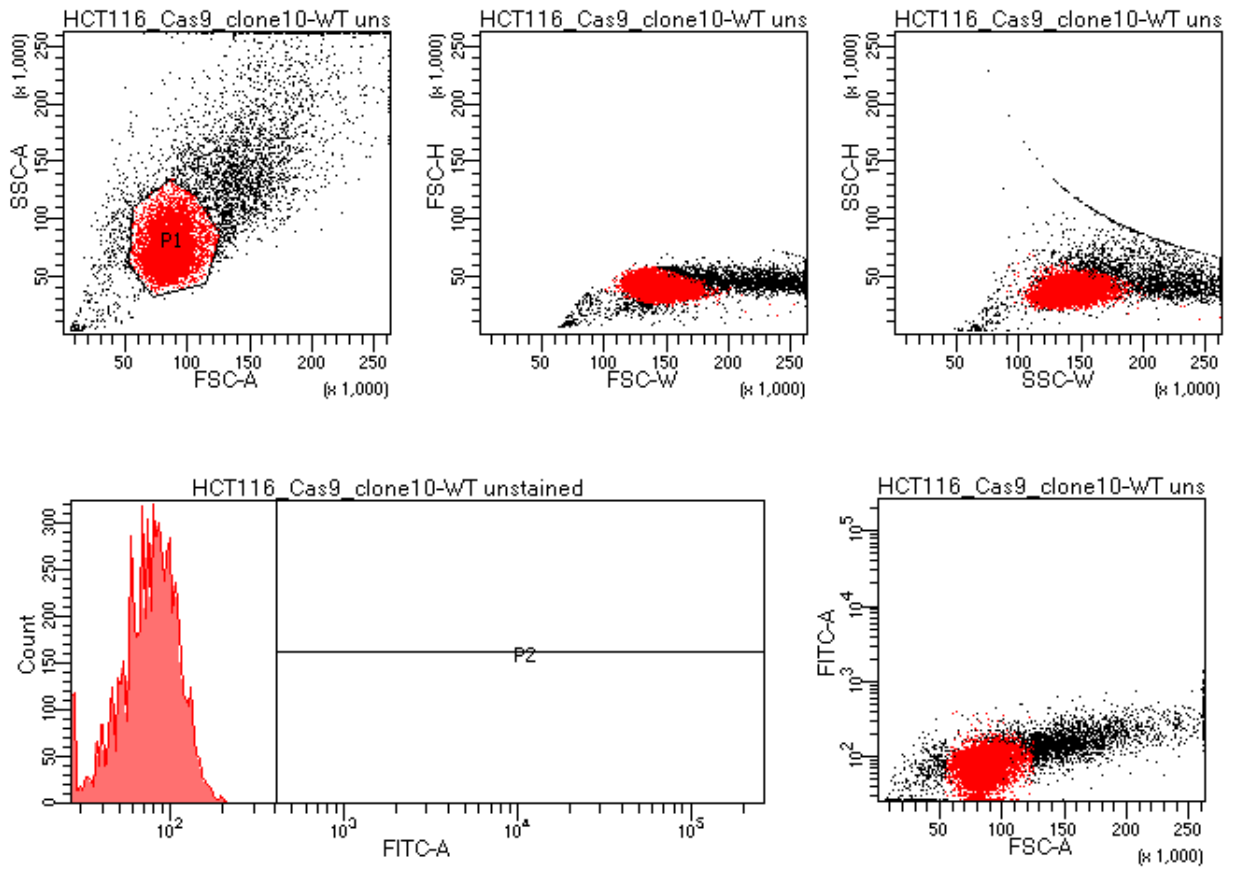

| Tube: WT unstained |         |         |        |
|--------------------|---------|---------|--------|
| Population         | #Events | %Parent | %Total |
| ■ All Events       | 10,000  | ####    | 100.0  |
| ■ P1               | 7,018   | 70.2    | 70.2   |
| ▣ P2               | 0       | 0.0     | 0.0    |

Supplement: Supplementary file 4 — Source Data [file 41467_2020_17221_MOESM4_ESM.zip › Source Data/SourceData_FACS/Figure 3c/HCT116_Cas9_clone10_WT unstained_01032016103714.pdf]

# BD FACSDiva 8.0.1

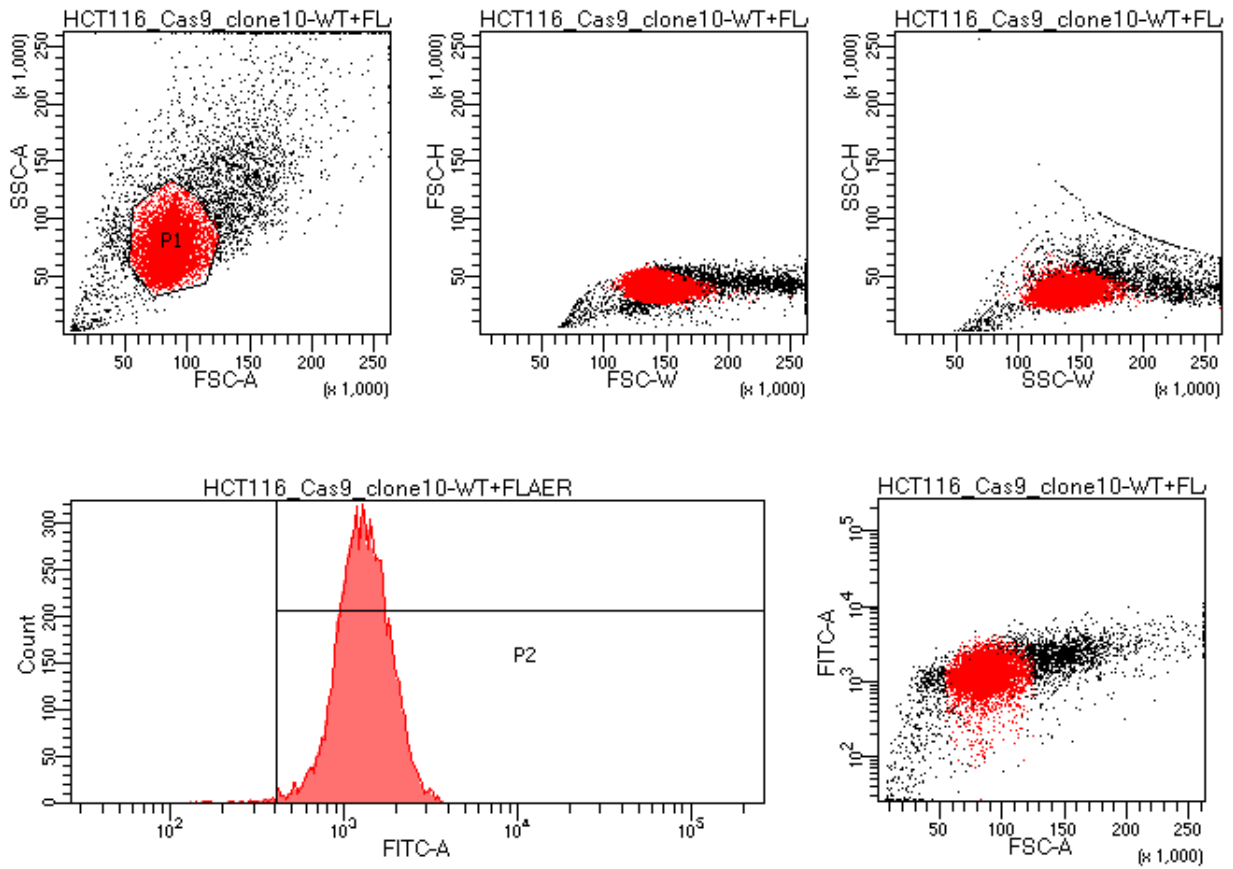

| Tube: WT+FLAER |         |         |        |
|----------------|---------|---------|--------|
| Population     | #Events | %Parent | %Total |
| ■ All Events   | 10,000  | ####    | 100.0  |
| ■ P1           | 7,528   | 75.3    | 75.3   |
| ▣ P2           | 7,396   | 98.2    | 74.0   |

Supplement: Supplementary file 4 — Source Data [file 41467_2020_17221_MOESM4_ESM.zip › Source Data/SourceData_FACS/Figure 3c/HCT116_Cas9_clone10_WT+FLAER_01032016103745.pdf]

# BD FACSDiva 8.0.1

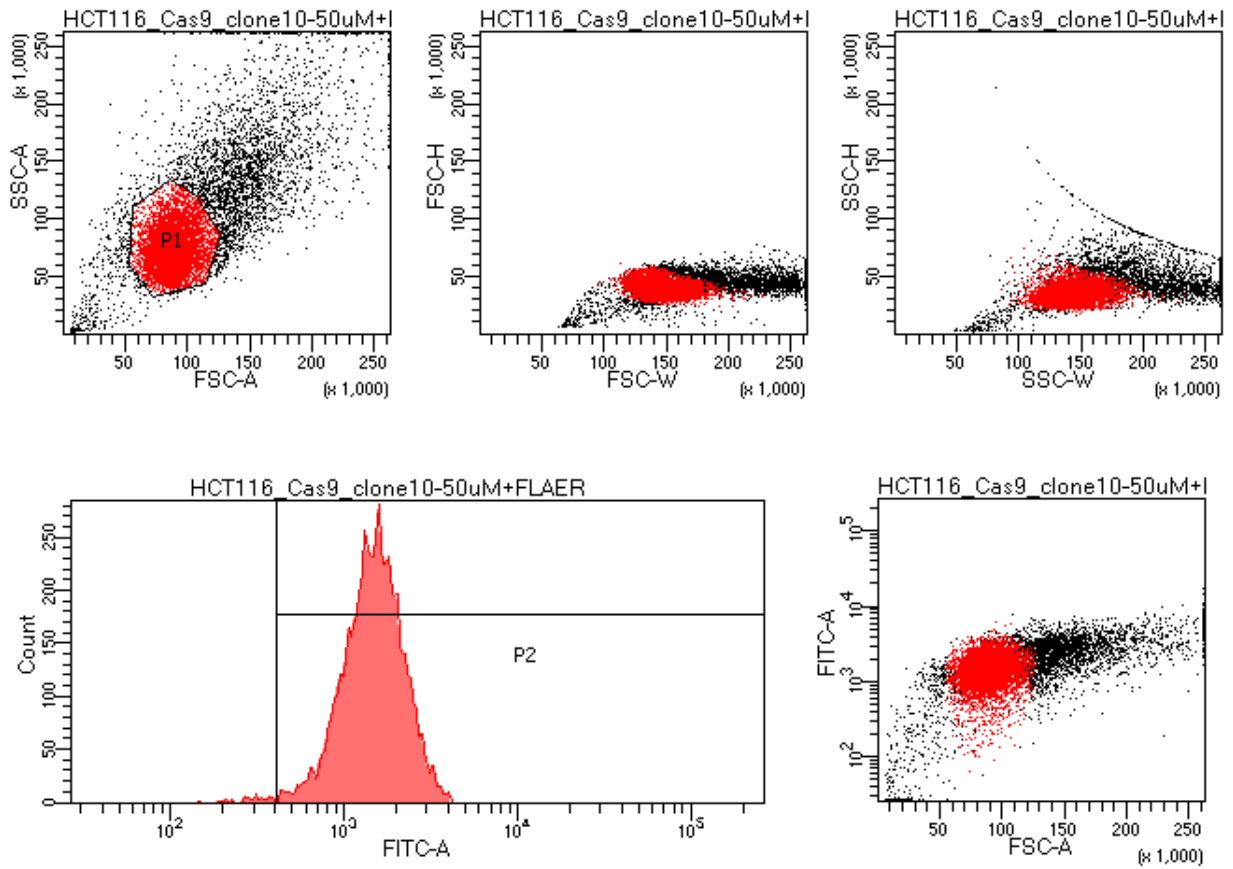

Tube: 50uM+FLAER

| Population   | #Events | %Parent | %Total |
|--------------|---------|---------|--------|
| ■ All Events | 10,000  | ####    | 100.0  |
| ■ P1         | 6,789   | 67.9    | 67.9   |
| ▣ P2         | 6,650   | 98.0    | 66.5   |

Supplement: Supplementary file 4 — Source Data [file 41467_2020_17221_MOESM4_ESM.zip › Source Data/SourceData_FACS/Figure 3c/HCT116_Cas9_clone10_50uM+FLAER_01032016103807.pdf]

# BD FACSDiva 8.0.1

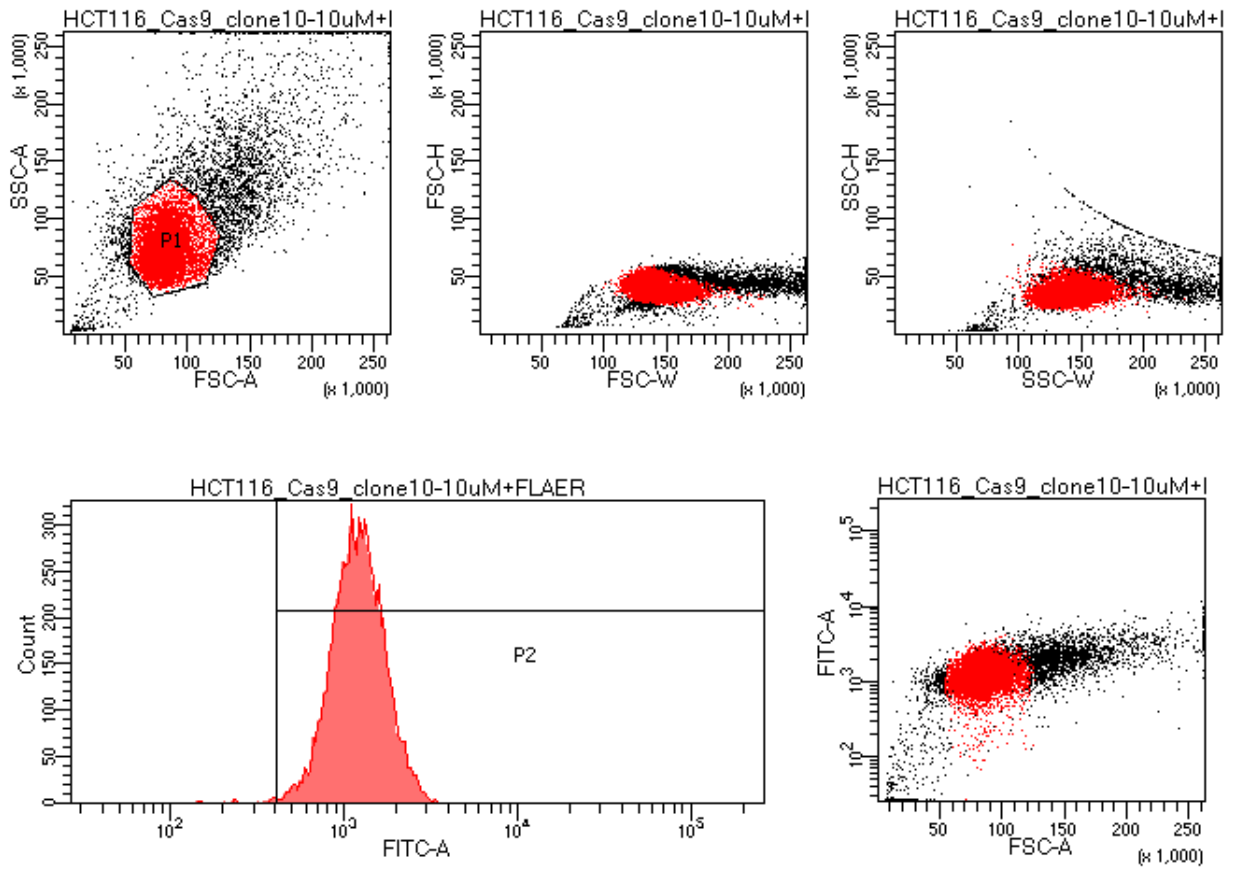

Tube: 10uM+FLAER

| Population   | #Events | %Parent | %Total |
|--------------|---------|---------|--------|
| ■ All Events | 10,000  | ####    | 100.0  |
| ■ P1         | 7,237   | 72.4    | 72.4   |
| ▣ P2         | 7,134   | 98.6    | 71.3   |

Supplement: Supplementary file 4 — Source Data [file 41467_2020_17221_MOESM4_ESM.zip › Source Data/SourceData_FACS/Figure 3c/HCT116_Cas9_clone10_10uM+FLAER_01032016103752.pdf]
